# *Drosophila septin interacting protein 1* regulates neurogenesis in the early developing larval brain

**DOI:** 10.1038/s41598-021-04474-3

**Published:** 2022-01-07

**Authors:** Jia-Yi Wei, Sao-Yu Chu, Yu-Chien Huang, Pei-Chi Chung, Hung-Hsiang Yu

**Affiliations:** Institute of Cellular and Organismic Biology, Academia Sinica, Taipei, Taiwan

**Keywords:** Cell biology, Developmental biology, Genetics, Neuroscience, Stem cells

## Abstract

Neurogenesis in the *Drosophila* central brain progresses dynamically in order to generate appropriate numbers of neurons during different stages of development. Thus, a central challenge in neurobiology is to reveal the molecular and genetic mechanisms of neurogenesis timing. Here, we found that neurogenesis is significantly impaired when a novel mutation, *Nuwa*, is induced at early but not late larval stages. Intriguingly, when the *Nuwa* mutation is induced in neuroblasts of olfactory projection neurons (PNs) at the embryonic stage, embryonic-born PNs are generated, but larval-born PNs of the same origin fail to be produced. Through molecular characterization and transgenic rescue experiments, we determined that *Nuwa* is a loss-of-function mutation in *Drosophila septin interacting protein 1* (*sip1*). Furthermore, we found that SIP1 expression is enriched in neuroblasts, and RNAi knockdown of *sip1* using a neuroblast driver results in formation of small and aberrant brains. Finally, full-length SIP1 protein and truncated SIP1 proteins lacking either the N- or C-terminus display different subcellular localization patterns, and only full-length SIP1 can rescue the *Nuwa*-associated neurogenesis defect. Taken together, these results suggest that SIP1 acts as a crucial factor for specific neurogenesis programs in the early developing larval brain.

## Introduction

Ensembles of neurons are produced by a limited number of neural stem cells (called neuroblasts in *Drosophila*) and assemble into complex neural circuits. These circuits comprise the functional nervous system required for animal survival and reproduction. The *Drosophila* nervous system is a well-characterized model that is widely used for investigating the molecular and genetic programs crucial for neurogenesis. In the adult *Drosophila* central brain, each hemisphere is composed of neurons from around 100 neuroblast-derived lineages and has a total number of roughly 11,000 neurons^1–3^. Notably, a previous study showed that the final neuronal composition (i.e., cell numbers and subtypes of neurons) for two olfactory neural lineages-anterodorsal projection neurons (adPNs) and lateral antennal lobe neurons-is extremely difficult to perturb, even under the harsh challenge of dietary protein starvation^4^. In contrast, the neurogenesis of certain groups of neurons like Kenyon cells, the intrinsic neurons of the learning and memory center (the mushroom body), is highly plastic and uncoupled from organismal growth and development^4^. Tight regulation, robustness and plasticity are key characteristics of neurogenesis that enable an organism to produce a functional nervous system. Therefore, revealing the molecular and genetic mechanisms that ensure generation of appropriate neuronal numbers and subtypes is an important but challenging task for neurobiologists.

Neurogenesis of the *Drosophila* central brain occurs during two major developmental periods^5^. At the embryonic stage, neuroblasts divide 10–20 times to produce the neurons that construct larval-specific neural circuits. Then, at postembryonic stages, the neuroblasts undergo 100–200 rounds of the cell cycle to produce more neurons; these postembryonic neurons are assembled with some of the embryonic-born neurons to constitute adult-specific neural circuits. Between these two waves of neurogenesis, neuroblasts undergo cell cycle arrest (at the end of embryogenesis) and resume proliferation at the early larval stage^5^. Interestingly, the cell bodies of quiescent neuroblasts become enlarged prior to their awakening and re-entry into the cell cycle; this reactivation process is regulated by Hippo, insulin receptor, and target of rapamycin signaling pathways at the early larval stage^6–8^. After reactivation of quiescent neuroblasts, the rates of neurogenesis for many neural lineages are accelerated, as evidenced by increased EdU/BrdU incorporation in the brain at early to mid-larval stages^9,10^. Most proliferative neuroblasts eventually lose their ability to divide at the early pupal stage, likely due to a switch in energy metabolism induced by the steroid hormone, ecdysone, and the Mediator complex. This switch results in the shrinkage of neuroblast cell body size and gradually leads neuroblasts to the exit the cell cycle^11^. Since neurogenesis is a protracted process with dynamic rates of neuron production, it is probable that specific molecular and genetic programs govern neurogenesis in a developmental stage-dependent manner, e.g., differential activities at early versus late larval stages.

In our ongoing MARCM (mosaic analysis with a repressible cell marker)^12^-based genetic screen for modulators of neurogenesis, we identified a novel mutation, *Nuwa*. In this mutant line, we found impaired neurogenesis in all examined neural lineages when the homozygous mutation was induced at the early larval but not embryonic or late larval stages. Interestingly, the gene defect responsible for the *Nuwa*-associated neurogenesis phenotype was mapped to *Drosophila septin interacting protein 1* (*sip1*)^13^. During development, SIP1 expression was enriched in neuroblasts and RNAi knockdown of *sip1* using a neuroblast driver could recapitulate *Nuwa*-associated aberrant brain features, including smaller brain size. Finally, we found that full-length SIP1 protein and truncated SIP1 proteins displayed preferential subcellular localizations, and only full-length SIP1 protein could rescue the *Nuwa*-associated neurogenesis defects. Taken together, these results suggest that SIP1 acts as a crucial factor for neurogenesis processes in the early developing larval brain.

## Results

### Induction of the ***P***^***111477***^ mutation at the early larval stage significantly impairs vPN neurogenesis

**Figure 1:**
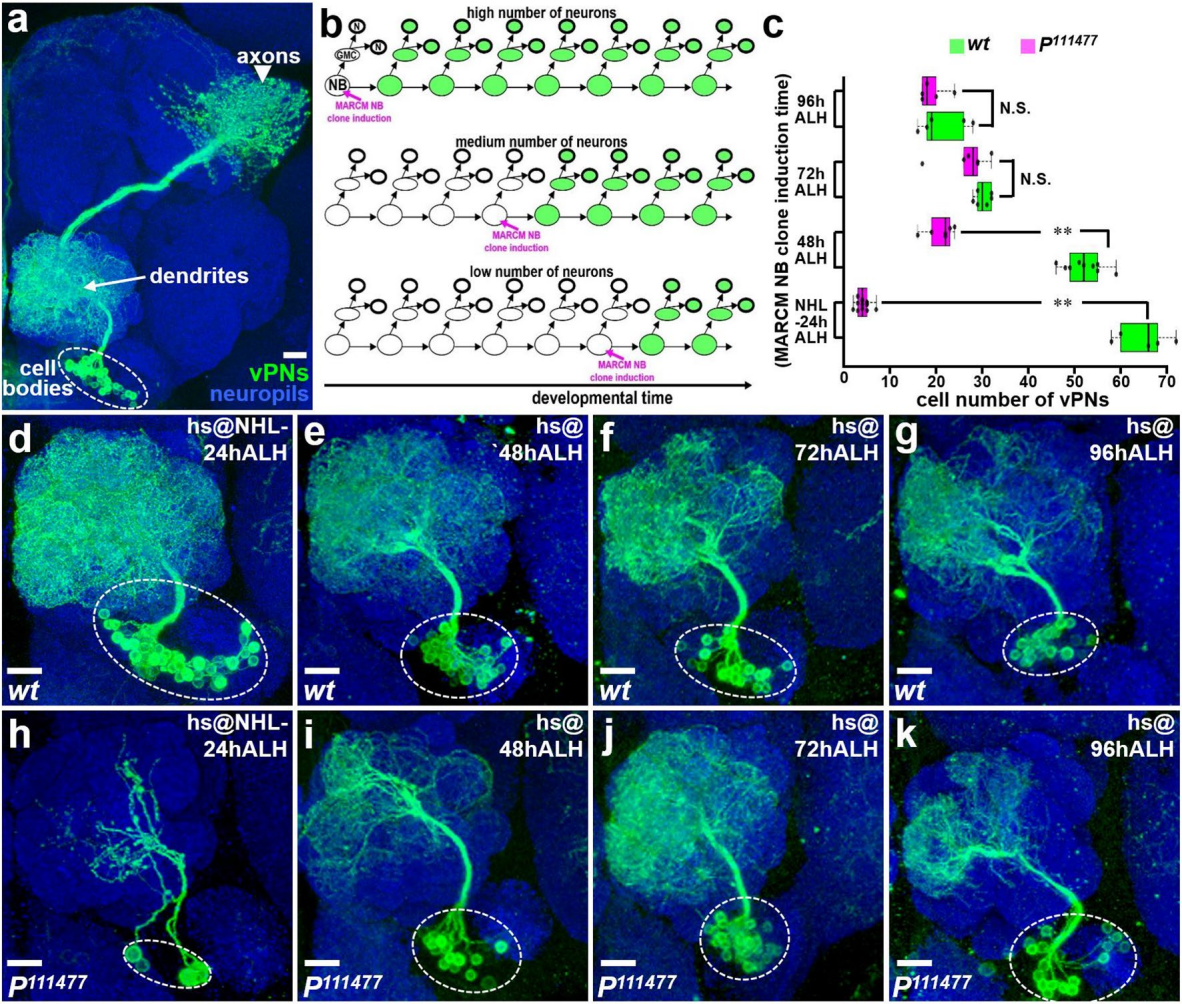
Neurogenesis of vPNs is significantly impaired when the *P*^*111477*^ mutation is induced at early but not late larval stages. (**a**) An example of a MARCM neuroblast (NB) clone is used to reveal the morphology of vPNs in the adult brain; using *GAL4-MZ699*. Axons (arrowhead) were primarily projected to the lateral horn, while dendrites (arrow) were found within the AL, and cell bodies (dashed circle) were distributed ventral to the AL. (**b**) Schematic drawings show predicted outcomes of MARCM clones induced at different developmental stages: high, medium and low numbers of neurons should be respectively observed when MARCM clones are induced at early, middle and late developmental stages. (**c**) Numbers of labeled vPNs were shown for wild-type (**d**–**g**) and *P*^*111477*^ mutants (**h**–**k**) when MARCM clones were induced at different larval stages. (**d**–**k**) Gradually reduced numbers of labeled vPNs (within the dashed circle) were observed in wild-type samples (**d**–**g**) when MARCM clones were induced at 24 h after larval hatching (NHL-24 h ALH), 48 h ALH, 72 h ALH and 96 h ALH. vPN neurogenesis was significantly impaired in the *P*^*111477*^ mutant samples (**h**–**k**) induced at early but not late larval stages*.* The genotypes shown in all figures are summarized in Supplemental table 1. Neuropils were revealed by the Bruchpilot (Brp) staining (blue). Scale bar: 10 μm.

We conducted a MARCM-based screen using *GAL4-MZ699*, which labels most ventral olfactory projection neurons (vPNs). With this ongoing screen we seek to identify mutations that affect vPN morphologies and hopefully provide clues about the genetic and molecular mechanisms underlying *Drosophila* central brain development (Fig. 1a). In our MARCM experiments, the labeling of *GAL4-MZ699*-positive vPNs is dependent on the induction of FRT (flippase recognition target)-mediated mitotic recombination in the vPN neuroblasts. Therefore, a gradual reduction in the number of labeled vPNs is expected when MARCM neuroblast clones are induced from early to late developmental stages (Fig. 1b). In agreement with this expectation, cell numbers of vPNs in wild-type flies were counted as 64.8 ± 5.8 when MARCM neuroblast clones were induced at newly hatched larvae to 24 h after larval hatching (NHL-24 h ALH; n = 5), 52.1 ± 4.1 at 48 h ALH (n = 9), 30.2 ± 1.7 at 72 h ALH (n = 6), and 21.4 ± 5.3 at 96 h ALH (n = 5) (Fig. 1c-g; Supplemental Fig. 1). However, we found that the number of vPNs was drastically reduced in the homozygous mutation caused by a P-element insertion line (Kyoto *Drosophila* Genome Research Center/DGRC 111,477, referred to as *P*^*111477*^) when MARCM neuroblast clones were induced at NHL-24 h ALH (4.2 ± 1.3, n = 14, *P* < 0.01; Fig. 1c,h; Supplemental Fig. 1). To investigate whether the *P*^*111477*^ mutation generally impairs the production of vPNs, we also examined the vPN number in MARCM neuroblast clones induced at later developmental stages. Despite our finding that the labeled vPN number was still substantially lower in *P*^*111477*^ mutant samples compared to wild-type samples when MARCM neuroblast clones were induced at 48 h ALH (21 ± 3, n = 6, *P* < 0.01; Fig. 1c,i; Supplemental Fig. 1), vPN neurogenesis was partially restored when the *P*^*111477*^ mutation was induced at 48 h ALH compared to NHL-24 h ALH (*P* < 0.01; ; Supplemental Fig. 1). In contrast, we were surprised to observe similar numbers of labeled vPNs in *P*^*111477*^ mutant and wild-type samples when MARCM neuroblast clones were induced at 72 h ALH (26.7 ± 5.2, n = 6, *P* > 0.5; Fig. 1c,j; Supplemental Fig. 1) or at 96 h ALH (19.2 ± 2.9, n = 5, *P* > 0.5; Fig. 1c,k; Supplemental Fig. 1). Taken together, these results suggested that the *P*^*111477*^ mutation compromised vPN neurogenesis at early but not late larval stages.

### Neurogenesis is compromised in various brain regions when the *Nuwa* mutation is induced at early but not late larval stage

Based on DGRC annotations, *P*^*111477*^ carries two P-element insertions, *l(2)k07109a* and *l(2)k07109b* (referred to as *P*^*07109a*^ and *P*^*07109b*^, respectively) on the *FRT*^*40A*^ background, which allows for MARCM-related experiments. *P*^*07109a*^ is inserted into an unknown gene at cytolocation 25F2, whereas *P*^*07109b*^ is inserted in the *Fas3* gene at cytolocation 36F2. To investigate whether loss of *Fas3* function is responsible for the neuronal production defect, we examined the vPN number in an independent *Fas3* mutant (DGRC 111,717, referred to as *P*^*111717*^). However, we found a normal number of vPNs arose from the *P*^*111717*^ mutant neuroblast clones, suggesting that the absence of *Fas3* alone did not compromise the production of vPNs (Supplemental Fig. 2a). In addition, we utilized a P-element insertion line (DGRC 102,523, referred to as *P*^*102523*^), which is annotated as a single *P*^*07109a*^ insertion without the *P*^*07109b*^ insertion, to test whether the vPN neurogenesis defect is caused by the *P*^*07109a*^ insertion. We assembled *P*^*102523*^ into the *FRT*^*40A*^ background, and as expected, the production of vPNs was impaired in the mutant. As such, the mutant had significantly fewer vPNs (*P*^*102523*^ mutant samples: 3.8 ± 1.1, n = 5, *P* < 0.01; Supplemental Fig. 2b,d) than wild-type samples when MARCM neuroblast clones were induced at NHL-24 h ALH. In contrast, vPN neurogenesis was relatively normal, and similar vPN numbers were found in wild-type and *P*^*102523*^ mutant samples (24 ± 4.4, n = 3, *P* > 0.1; Supplemental Fig. 2c,d) when MARCM neuroblast clones were induced at 72 h ALH. Since these results together suggested that *P*^*07109a*^ is probably the mutation in *P*^*111477*^ that compromises vPN neurogenesis, the *P*^*102523*^ mutation was used in most of the subsequent experiments.

**Figure 2:**
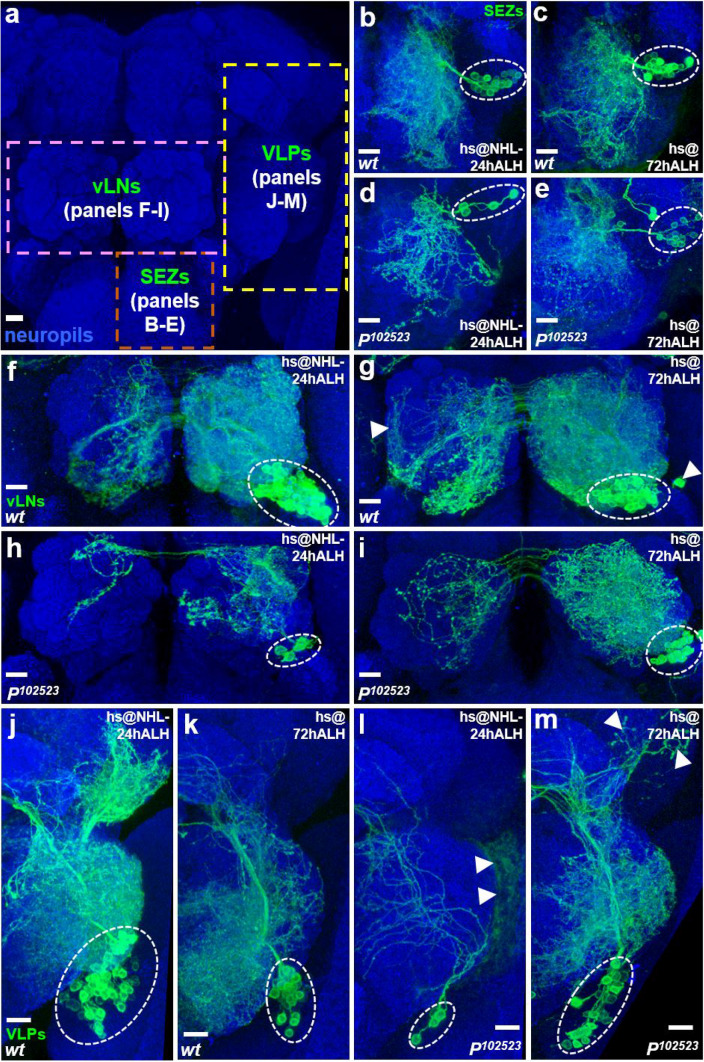
Neurogenesis is generally compromised in various brain regions when the *Nuwa* mutation is induced at the early but not late larval stages. (**a**) Three groups of neurons were used to examine the developmental stage-dependent requirement for *Nuwa*, including neurons in the subesophageal zone (SEZs; panels **b**–**e**), ventral olfactory interneurons in the AL (vLNs; panels **f**–**i**) and neurons in the ventrolateral protocerebrum (VLPs; panels J-M). (**b**–**m**) All three groups of neurons displayed severe neurogenesis defects with the *P*^*102523*^ (*Nuwa*) mutation when MARCM clones were induced at NHL-24 h ALH (panels **b**, **d**, **f**, **h**, **j**, **l**), whereas no obvious neurogenesis defects were observed in neurons with the *Nuwa* mutation when MARCM clones were induced at 72 h ALH (panels **c**, **e**, **g**, **i**, **k**, **m**). Neuropils were revealed by the Brp staining (blue), and background neurons are indicated by arrowheads. Scale bar: 10 μm.

We wondered whether the neurogenesis defect caused by the *P*^*102523*^ mutation is restricted to vPNs or if it broadly occurs in other groups of neurons. Therefore, we examined neurons generated in various brain regions (Fig. 2a), including neurons in the subesophageal zone (SEZs; Fig. 2b–e), ventral olfactory interneurons in the AL (vLNs; Fig. 2f–i) and neurons in the ventrolateral protocerebrum (VLPs; Fig. 2j–m). Notably, all of the examined neurons displayed severe neurogenesis defects in the *P*^*102523*^ mutation when MARCM neuroblast clones were induced at NHL-24 h ALH (Fig. 2b,d,f,h,j,l). On the other hand, we did not observe obvious neurogenesis defects among any examined groups of neurons in the *P*^*102523*^ mutation when MARCM neuroblast clones were induced at 72 h ALH (Fig. 2c,e,g,i,k,m). Of note, the neuron morphologies were generally aberrant in *P*^*102523*^ mutant clones for all neuronal groups examined in Figs. 1 and 2, implying a possible second role of *Nuwa* in neuronal morphogenesis. Since we aimed to focus this study on delineating the role of *Nuwa* in neurogenesis, we did not investigate the putative role of *Nuwa* in neuronal morphogenesis during development. Taken together, the results thus far suggested that the gene disrupted by the *P*^*102523*^ insertion is generally required for neurogenesis in various brain regions at the early but not late larval stages. This function of the unknown gene reminded us of the legend of an ancient goddess, Nuwa, who is considered to be the creator of mankind in Chinese myths. Therefore, we named the mutation *Nuwa* to reflect its essential role in the control of neurogenesis at the early larval stage.

### Embryonic-born adPNs are produced normally when the *Nuwa* mutation is induced at the embryonic stage but postembryonic-born adPNs are not

**Figure 3:**
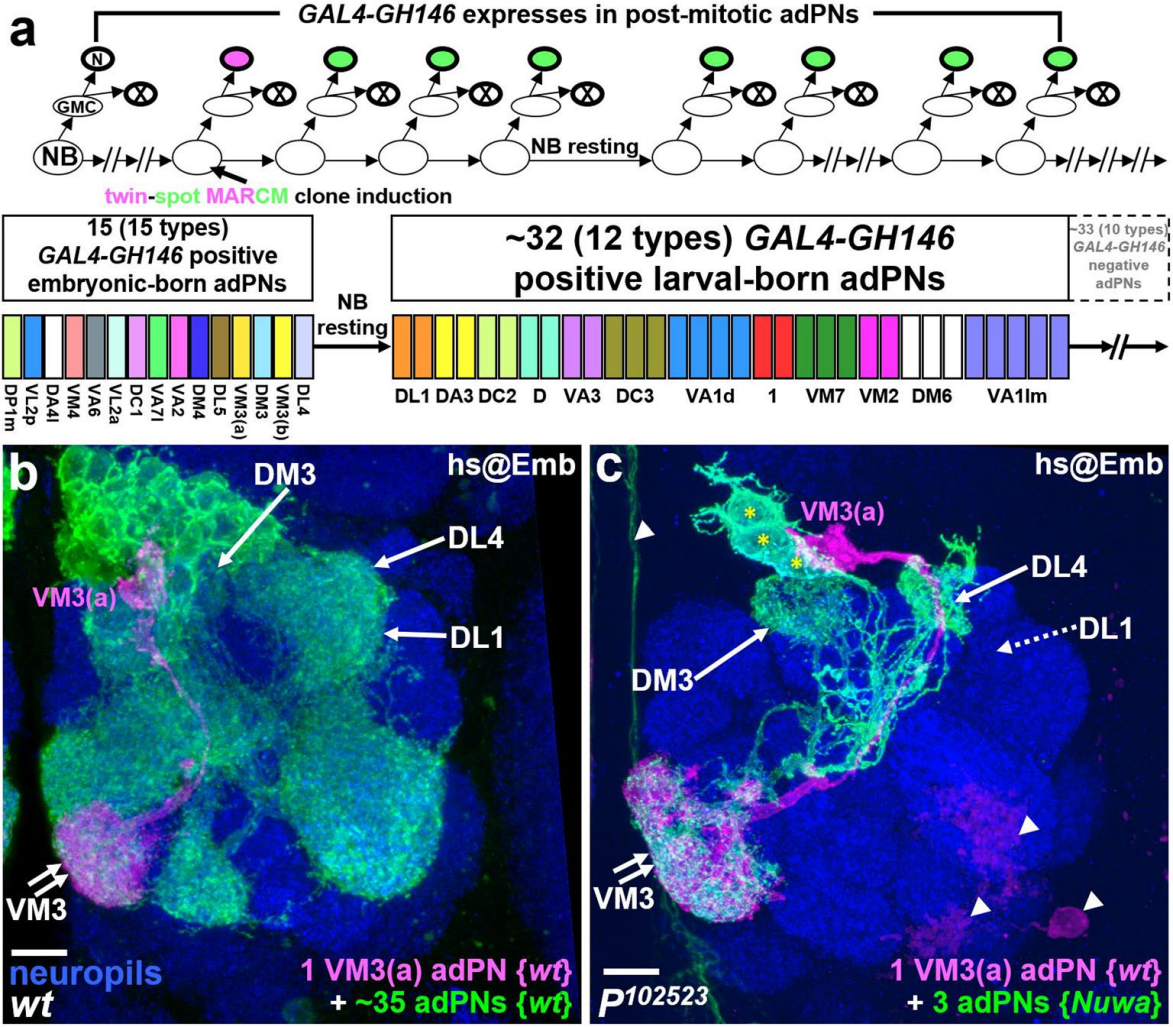
Embryonic-born adPNs are produced normally but postembryonic-born adPNs fail to appear when the *Nuwa* mutation is induced at the embryonic stage. (**a**) Schematic drawings show a predicted outcome for a twin-spot MARCM clone when it is induced at the embryonic stage (upper panel); the known cell numbers and subtypes of embryonic- and larval-born adPNs labeled by *GAL4-GH146* are shown (bottom panel). (**b**, **c**) Examples of wild-type and *Nuwa* mutant twin-spot MARCM clones induced at the embryonic stage. In the wild-type sample (panel **b**), a VM3a adPN (an embryonic-born adPN in magenta) was associated with around 35 adPNs (containing both embryonic- and larval-born adPNs in green); in the *P*^*102523*^ (*Nuwa*) mutant (panel **c**), a VM3a wild-type adPN (magenta) was associated with three *Nuwa* mutant adPNs (green, marked by asterisks). Dendrites of the three green *Nuwa* mutant adPNs were only observed in DM3, VM3 and DL4 [but not DL1 (indicated by dashed arrow)] glomeruli of the AL, indicating that they belong to the last three types of embryonic-born adPNs and further suggesting that no larval-born adPNs were generated*.* Neuropils were revealed by Brp staining (blue), and background neurons are indicated by arrowheads. Scale bar: 10 μm.

Since neurogenesis in the *Drosophila* central brain occurs at both embryonic and postembryonic stages, we also wondered whether *Nuwa* dictates neurogenesis at times other than the early larval stage. In particular, we wondered whether *Nuwa* is also required for neurogenesis at the embryonic stage. Since adPNs are well-characterized in terms of neuronal numbers and subtypes produced at both embryonic and postembryonic stages, we focused on adPNs to assess the requirement of *Nuwa* at the embryonic stage^14^. First, we confirmed that a neurogenesis defect was indeed present in adPNs when the *Nuwa* mutation was induced at the early larval stage (Supplemental Fig. 3). We then conducted twin-spot MARCM experiments using *GAL4-GH146*, which labels 15 types (with a total cell number of 15) of embryonic-born adPNs as well as the first 12 types (with a total cell number of around 32) of larval-born adPNs, to comprehensively analyze the *Nuwa*-associated neurogenesis defect in adPNs^14^ (Fig. 3a). In wild-type animals, a VM3a adPN (an embryonic-born adPN), which was associated with around 35 adPNs (both embryonic- and larval-born adPNs), was labeled when a twin-spot MARCM clone was induced at the embryonic stage (Fig. 3b). In contrast, a VM3a wild-type adPN associated with three *Nuwa* mutant adPNs was seen in a twin-spot MARCM clone when the twin-spot MARCM clone was induced at a similar embryonic stage (Fig. 3c). Notably, the dendrites of these three green *Nuwa* mutant adPNs were arborized in DM3, VM3 and DL4 glomeruli of the AL (Fig. 3c). This arborization pattern implied that the cells were the last three types of embryonic-born adPNs and further suggested that no larval-born adPN was generated when *Nuwa* was mutated in adPNs at the embryonic stage. Moreover, additional analyses of twin-spot MARCM clones derived from the *Nuwa* mutant adPN neuroblast all led to a similar conclusion, i.e., the generation of embryonic-born adPNs was unaffected, but production of larval-born adPNs was prevented when adPN neuroblasts became *Nuwa* mutants at the embryonic stage (Supplemental Fig. 4). Collectively, our data showed that neurogenesis of embryonic-born, but not larval-born, adPNs was relatively normal when the *Nuwa* mutation was induced starting from the embryonic stage in twin-spot MARCM experiments.

**Figure 4:**
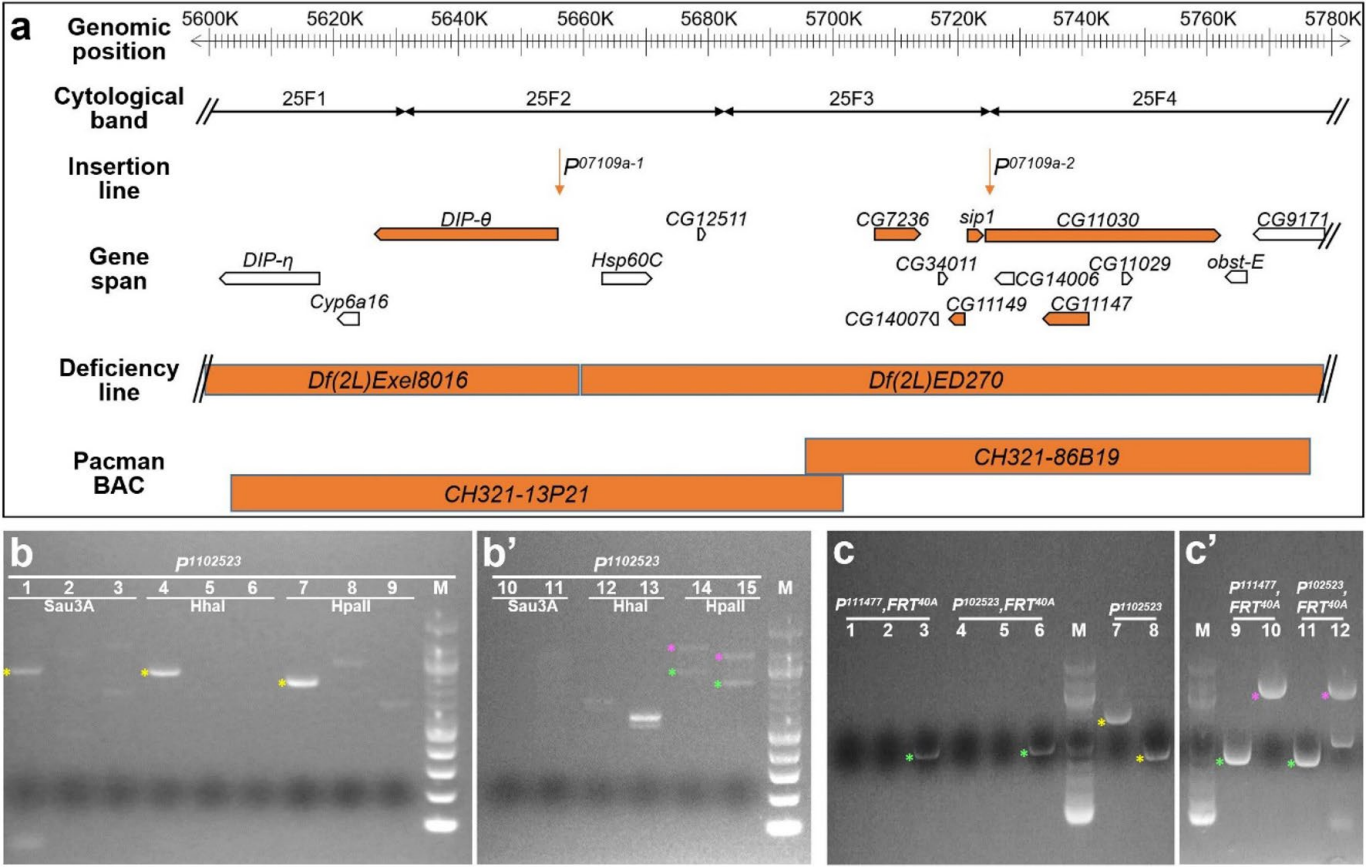
Molecular characterization of *Nuwa*. (**a**) Illustration shows genomic position, cytological bands, P-element insertion lines, gene span, deficiency lines and Pacman genomic BAC clones related to *Nuwa*. Reagents tested in this study are shown in orange. (**b**, **b**ʹ) Genomic DNA isolated from the *P*^*102523*^ insertion line was cut with restriction enzymes (e.g., Sau3A, HhaI and HpaII), self-ligated into circularized DNAs and then used as a template for inverse PCR. The PCR products contained flanking DNA fragments of *P*^*102523*^, which were mapped to three genes, including *Fas3* (yellow asterisks; lanes 1, 4 and 7), *DIP-θ* (green asterisks; lanes 14 and 15) and *CG11030* (magenta asterisks; lanes 14 and 15). (**c**, **c**ʹ) Genomic DNA isolated from *P*^*102523*^, *P*^*111477*^, *FRT*^*40A*^ and *P*^*102523*^, *FRT*^*40A*^ mutant lines was directly used for PCR reactions to obtain DNA fragments flanking P-element insertions. P-element-flanking DNA fragments mapped to *Fas3* (yellow asterisks) in genomic DNA from the *P*^*102523*^ mutant line (lanes 7 and 8), but not in *P*^*111477*^, *FRT*^*40A*^ (lanes 1 and 2) or *P*^*102523*^, *FRT*^*40A*^ (lanes 4 and 5) mutant lines. In contrast, P-element-flanking DNA fragments mapped to *DIP-θ* (green asterisks; lanes 3, 6, 9 and 11) and *CG11030* (magenta asterisks; lanes 10 and 12) were found in genomic DNA isolated from *P*^*111477*^, *FRT*^*40A*^ and *P*^*102523*^, *FRT*^*40A*^ mutant lines. Oligos used for reverse PCR and PCR reactions in panels **b**, **b**ʹ, **c**, **c**ʹ are listed in Supplemental table 3.

### Molecular characterization and identification of the *Nuwa* mutation as a loss-of-function in *Drosophila septin interacting protein 1*

To identify the gene that is interrupted by the insertion of *P*^*07109a*^ and causes the *Nuwa*-associated neurogenesis phenotype, we used an inverse PCR method^15^ to identify the genomic DNA fragments flanking *P*^*07109a*^ (Fig. 4a). However, we encountered two unexpected results after recovering P-element-flanking genomic DNA fragments from *P*^*102523*^, *P*^*111477*^, *FRT*^*40A*^ and *P*^*102523*^, *FRT*^*40A*^ mutant lines. First, the *P*^*07109b*^ insertion still appeared to be present in the original *P*^*102523*^ mutant line, since a genomic DNA fragment flanking the P-element was matched to *Fas3* (Fig. 4b,c; Supplemental Fig. 5). In contrast, the *P*^*07109b*^ insertion was not identified in *P*^*111477*^, *FRT*^*40A*^ or *P*^*102523*^, *FRT*^*40A*^ mutant lines, since we did not recover P-element-flanking DNA fragments for *Fas3* from these lines (Fig. 4c; Supplemental Fig. 5). In retrospect, it probably should not have been overly surprising that *P*^*07109b*^ could be lost in the process of performing genetic crosses to generate *P*^*111477*^, *FRT*^*40A*^ and *P*^*102523*^, *FRT*^*40A*^ flies because *P*^*07109b*^ is located between *FRT*^*40A*^ and *P*^*07109a*^. The second unexpected result was that *P*^*07109a*^ in *P*^*111477*^, *FRT*^*40A*^ and *P*^*102523*^, *FRT*^*40A*^ mutant lines consisted of P-elements inserted into two different genes, *Dpr-interacting protein θ* (*DIP-θ*; *P*^*07109a-1*^ is inserted 76 bp downstream of the beginning of the *DIP-θ* transcript at cytolocation 25F2) and *CG11030* (*P*^*07109a-2*^ is inserted 21 bp downstream of the beginning of the *CG11030* transcript at cytolocation 25F4) (Fig. 4a–c). Based on this information, we further investigated whether loss of *CG11030* or *DIP-θ* function is responsible for the *Nuwa*-associated neurogenesis defect.

**Figure 5:**
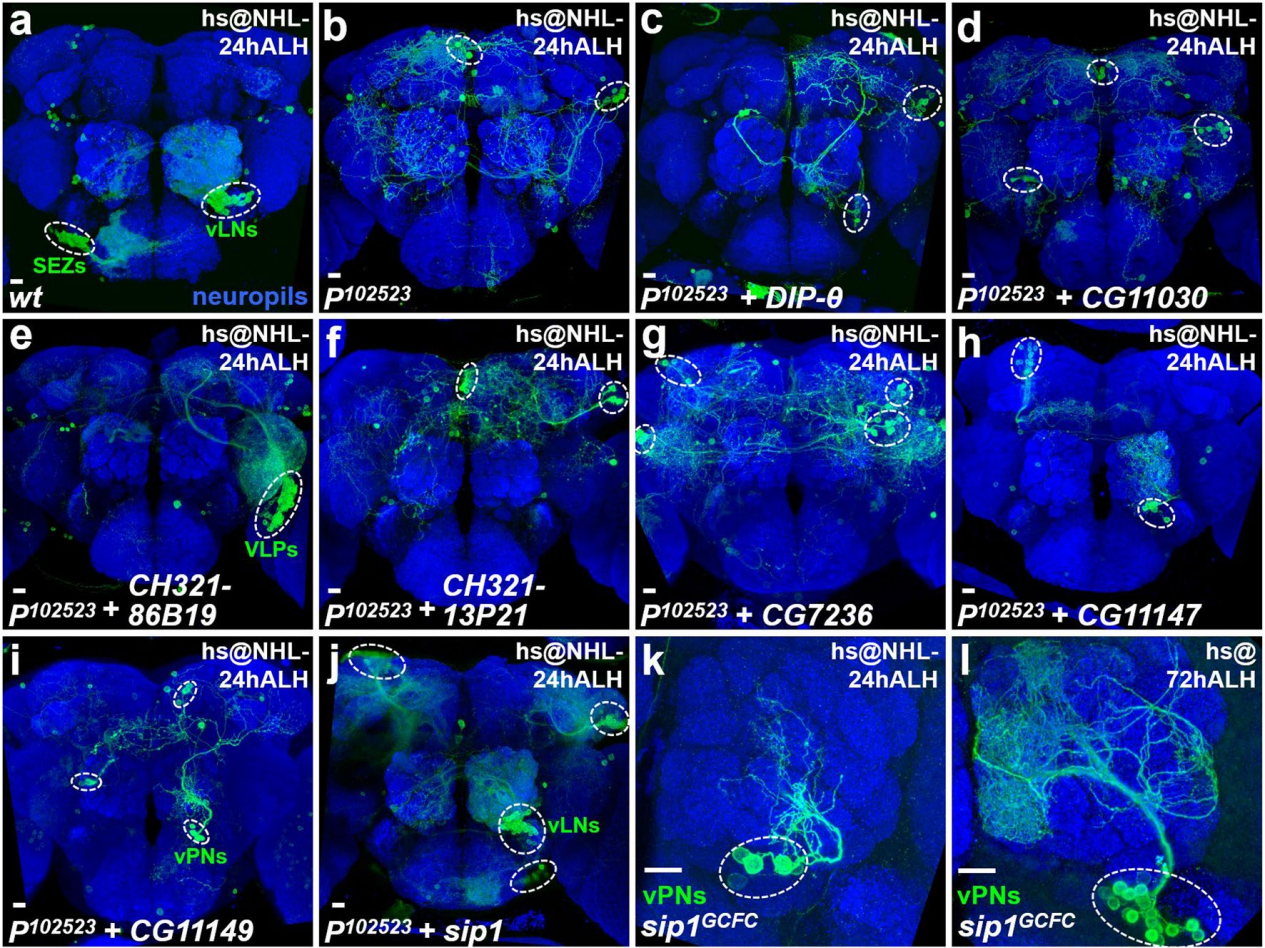
The *Nuwa* mutation maps as a loss-of-function of *sip1*. (**a**–**d**) Overexpression of the *DIP-θ* or *CG11030* cDNA transgene driven by *Act-FRT* < *stop* < *FRT-GAL4* failed to rescue the neurogenesis defect in *P*^*102523*^ mutants. All wild-type neural lineages (with cell numbers and morphology) labeled by *Act-FRT* < *stop* < *FRT-GAL4* can be found in the previous study (Yu et al., 2013; reference #3). (**e**–**f**) The *Nuwa*-associated neurogenesis defect in the *P*^*102523*^ mutation was rescued by the *CH321-86B19* BAC genomic clone, but not the *CH321-13P21* BAC genomic clone. (**g**–**i**) Overexpression of *CG7236*, *CG111477* and *CG11149* cDNA transgenes driven by *Act-FRT* < *stop* < *FRT-GAL4* still failed to rescue the *Nuwa*-associated neurogenesis defect in the *P*^*102523*^ mutants. (**j**) In contrast, overexpression of the *sip1* transgene driven by *Act-FRT* < *stop* < *FRT-GAL4* significantly rescued the neurogenesis defect in the *P*^*102523*^ mutants. (**K** and **L**) The custom-made *sip1*^*GCFC*^ mutation recapitulated the *Nuwa*-associated neurogenesis defect*.* Neuropils were revealed by Brp staining (blue). Scale bar: 10 μm.

We overexpressed cDNA transgenes for *CG11030* or *DIP-θ* in *Nuwa* mutant MARCM neuroblast clones using a pan-cell driver (*Act-FRT* < *stop* < *FRT-GAL4*)^3^ and looked for rescue of the neurogenesis defect. However, neither *CG11030* nor *DIP-θ* was able to rescue the *Nuwa*-associated neurogenesis defect (Fig. 5a–d), suggesting that loss of *CG11030* or *DIP-θ* function alone may not cause the defect. In addition, these results raised a concern as to whether the *Nuwa*-associated neurogenesis defect could be derived from a background mutation in chromosome 2L, outside the genomic region of cytolocation 25F2-4, since the *Nuwa* phenotype was found in MARCM experiments using *FRT*^*40A*^.

To resolve this issue, we employed two approaches using deficiency and transgenic lines carrying bacterial artificial chromosome (BAC) genomic DNAs to map the genomic region of *Nuwa*. First, we selected two deficiency lines with deletions of the genomic region at cytolocation 25F1-4, including *Df(2L)Exel8016* (genomic region from the *DIP-η* gene to the *DIP-θ* gene is deleted) and *Df(2L)ED270* (genomic region from the *Hsp60c* gene to the *CG9171* gene is deleted) (Fig. 4a). Since the homozygous mutation of *P*^*102523*^ insertion is lethal (Supplemental table 2), we performed a complementation test to examine whether animals can survive when carrying trans-heterozygous mutations of *P*^*102523*^ with *Df(2L)Exel8016* or *Df(2L)ED270*. Interestingly, we found that trans-heterozygous mutations of *P*^*102523*^ with *Df(2L)ED270*, but not *Df(2L)Exel8016*, were lethal (Supplemental table 2). Thus, we concluded that the putative *Nuwa* mutation can be found within the genomic region deleted in *Df(2L)ED270* but not *Df(2L)Exel8016*. We then recombined *Df(2L)ED270* to the *FRT*^*40A*^ background and conducted MARCM experiments to examine whether *Df(2L)ED270* affects neurogenesis like the *P*^*102523*^ mutation. Indeed, we found that the *Df(2L)ED270* mutant neuroblast clones displayed neurogenesis defects similar to those in *Nuwa* mutants. Substantially fewer vPNs were produced when MARCM neuroblast clones were induced at NHL-24 h ALH, and relatively normal numbers were observed when induction was at 72 h ALH (Supplemental Fig. 6). These results strongly suggested that the *Nuwa* mutation resides in the genomic region of cytolocation 25F2-4.

**Figure 6:**
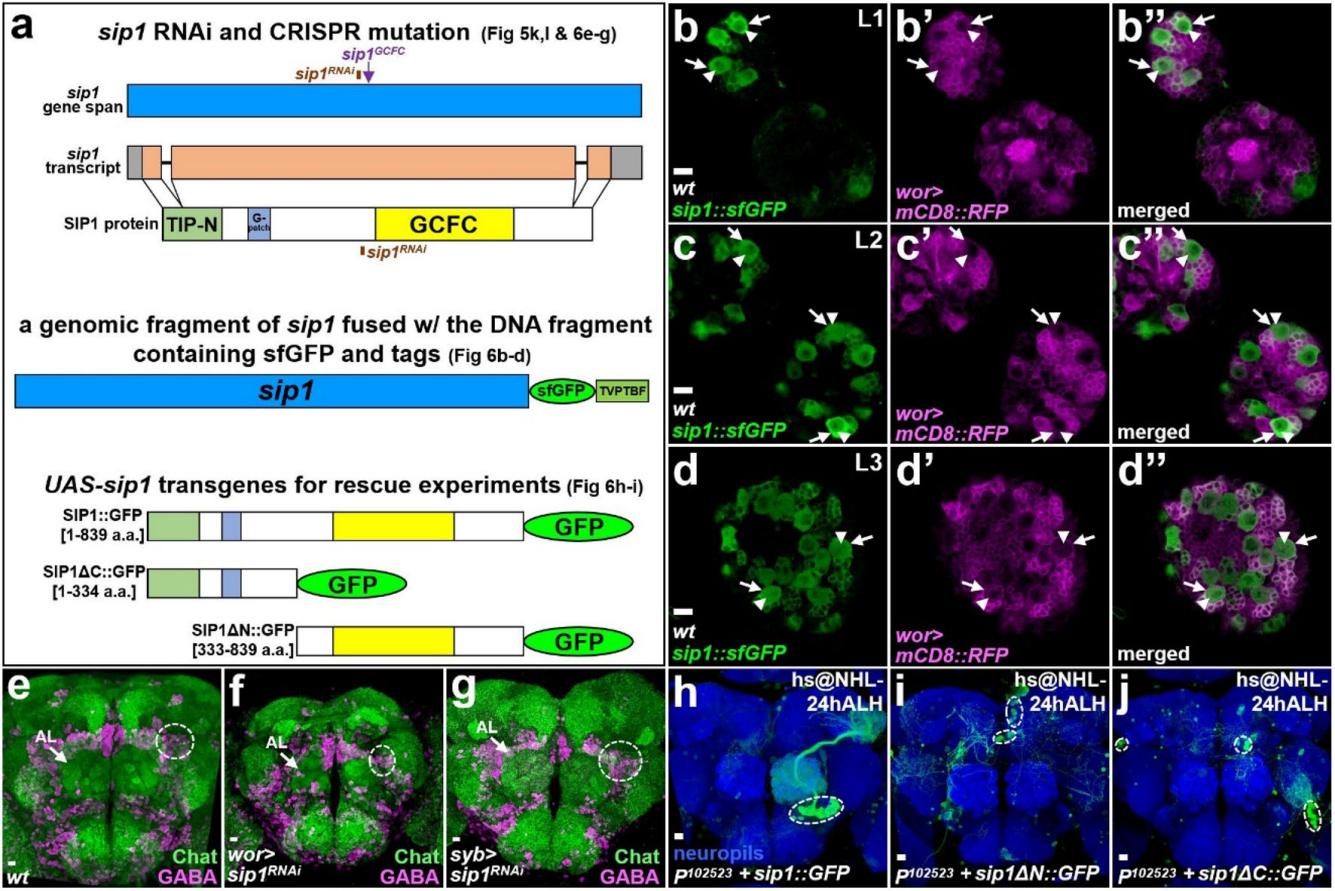
The SIP1 expression pattern, RNAi knockdown of *sip1* and SIP1:: GFP fusion proteins. (**a**) Schematic drawing shows the gene span, transcript, protein structure and reagents related to *sip1* (upper panel). TIP-N: Tuftelin-interacting protein N terminal domain; G-patch: domain enriched with highly conserved glycines; GCFC: domain containing a sequence similar to a GC-rich sequence DNA-binding factor, transcriptional repressor and histone-interacting proteins. Three SIP1:: GFP fusion proteins were used in this study, including the full-length SIP1:: GFP, SIP1ΔC:: GFP and SIP1ΔN:: GFP (bottom panel). (**b**–**d**) SIP1:: sfGFP expression (green; the transgenic fly was obtained from Vienna Drosophila Resource Center, VDRC318488) was enriched in neuroblasts. Estimation of the relative SIP1:: sfGFP expression level in neuroblast and neurons can be found in Supplemental Fig. 7. The plasma membrane (outer) and the nuclear membrane (inner) of neuroblasts are indicated by arrows and arrowheads, respectively (labeled in magenta by mCD8:: RFP driven by *worniu-GAL4* (*wor*)). (**e**–**g**) RNAi knockdown of *sip1* using a GAL4 line expressed in neuroblasts, *worniu-GAL4* (*wor*), but not using a pan-neuronal GAL4 line, *synaptobrevin-GAL4* (*syb*), resulted in aberrant brain morphology, including overall smaller brain size, abnormal neuropil architectures (the AL was indicated by arrows). Excitatory and inhibitory neurons were visualized by choline acetyltransferase (Chat; green) and γ-aminobutyric acid (GABA; magenta) staining. A reduction of GABA-positive neuronal number dorsolateral to the AL was observed in *wor* > *sip1*^*RNAi*^ knockdown samples (dashed circles; wild-type: 123.5 ± 13.3, n = 6; *wor* > *sip1*^*RNAi*^: 39.3 ± 11.3, n = 6; *syb* > *sip1*^*RNAi*^: 127.3 ± 24.3, n = 6). (**h**–**j**) The *Nuwa*-associated neurogenesis defect in the *P*^*102523*^ mutation was rescued by the full-length *sip1::GFP* transgene, but not *sip1ΔN::GFP* or *sip1ΔC::GFP* transgenes*.* Neuropils were revealed by Brp staining (blue). Scale bar: 10 μm.

In addition to the deficiency line experiments, we also made two customized transgenic lines carrying Pacman BAC genomic DNAs^16^, *CH321-13P21* (containing the genomic region of cytolocation 25F1-3, from the *DIP-θ* gene to the *CG12511* gene) and *CH321-86B19* (containing the genomic region of cytolocation 25F3-4, from the *CG7236* gene to the *CG9171* gene), to conduct rescue experiments with the *Nuwa* mutants (Fig. 4a). Intriguingly, the *Nuwa*-associated neurogenesis defect was rescued by the transgenic line carrying *CH321-86B19*, but not *CH321-13P21*, as a significantly higher number of neurons was generated by the *Nuwa* mutant neuroblast clones (Fig. 5e,f). Taken together, the experimental results from both deficiency lines and genomic transgenic rescue lines indicated that the *Nuwa* gene resides in the genomic region carried by *CH321-86B19* and deleted in the *Df(2L)ED270* line. Both of these conclusions rule out the possibility that *Nuwa* is a background mutation outside the genomic region of cytolocation 25F2-4.

Based on these deficiency and genomic DNA rescue results, we further generated additional cDNA transgenic lines carrying individual genes within *CH321-86B19* to identify the specific gene involved in the *Nuwa*-associated neurogenesis defect. Four out of 10 genes, *CG7236*, *CG11147*, *CG11149* and *septin interacting protein 1* (*sip1*) were initially examined for their abilities to rescue the *Nuwa*-associated neurogenesis defect (Figs. 4a and 5g,j). Three of the genes, *CG7236*, *CG11147* and *CG11149*, failed to rescue the *Nuwa*-associated neurogenesis defect when overexpressed in the *Nuwa* mutant neuroblast clones (Fig. 5g-i). On the other hand, overexpression of *sip1* significantly rescued the *Nuwa*-associated defect, as a substantial number of neurons were restored in *Nuwa* mutant neuroblast clones (Fig. 5j). To further test whether the loss of *sip1* function indeed causes the *Nuwa*-associated neurogenesis phenotype, we generated an insertion line, *sip1*^*GCFC*^. To create this line, we used CRISPR-Cas9^17,18^ to insert a DNA fragment that replaces part of the coding region of the *sip1* gene (Fig. 6a). As expected, we observed similar vPN neurogenesis defects in the *sip1*^*GCFC*^ mutation when MARCM neuroblast clones were induced at NHL-24 h ALH and 72 h ALH, respectively (Fig. 5k,l). Taken together, these results strongly suggested that the neurogenesis defect seen in the *Nuwa* mutation was due to the loss of *sip1* function.

### SIP1 expression is enriched in neuroblasts and *sip1* RNAi knockdown using a neuroblast driver causes brain defects

According to the predicted protein sequence and functional domain analysis, SIP1 contains 839 amino acids and at least three domains^13^ (Fig. 6a). The first domain is a Tuftelin-interacting protein N-terminal (TIP-N) domain at the N-terminus of SIP1, which has been shown to participate in enamel assembly by interacting with an enamel matrix protein, Tuftelin, in mice^19^. The next domain, called the G-patch domain, is enriched with highly conserved glycines; this type of domain has been found in a number of RNA binding proteins and has a putative function in RNA-related biological processes^19^. The third domain contains a sequence similar to GC-rich sequence DNA-binding factor, transcriptional repressor and histone-interacting proteins (GCFC), and it is presumably involved in transcriptional regulation^13,19^. To detect the distribution of SIP1 protein in the brain, we took advantage of a GFP-tagged transgenic line, *sip1::sfGFP* (obtained from Vienna Drosophila Resource Center, VDRC318488), generated from a genome-wide forsmid library containing tagged genes with mostly intact regulatory fragments^20^ (Fig. 6a). Interestingly, we found that the SIP1::sfGFP expression was enriched in neuroblasts during development (Fig. 6b–d; the relative expression level of SIP1::sfGFP in neuroblasts compared to that in neurons was estimated in Supplemental Fig. 7). Consistent with the SIP1::sfGFP expression pattern, RNAi knockdown of *sip1* using a neuroblast driver, *worniu-GAL4*, resulted in brain defects, including overall smaller brain size, abnormal neuropil architectures and a reduction of neuronal number (Fig. 6e,f; Supplemental Fig. 8a,b). In contrast, no obvious defects were observed when *sip1* expression was silenced using a differentiated neuronal driver, *synaptobrevin-GAL4* (Fig. 6g). Taken together, these results suggested that SIP1 indeed plays an important role in neurogenesis, and its function may be associated with neuroblasts at the larval stage.

### Truncated and full length SIP1 proteins show different subcellular localizations and only full-length SIP1 rescues the *Nuwa*-associated neurogenesis defect

To visualize the subcellular localization of SIP1 and gain insight into its functional domains, we generated three transgenic animals that expressed SIP1::GFP fusion proteins with different truncations (Fig. 6a). The full-length SIP1::GFP was constructed by fusing GFP to the C-terminus of full-length SIP1 (containing 839 amino acids), whereas SIP1ΔC::GFP and SIP1ΔN::GFP were made by fusing GFP to the C-terminus of two truncated SIP1 proteins carrying amino acids 1–334 and 333–839 of SIP1, respectively (Fig. 6a). Interestingly, all three SIP1::GFP fusion proteins displayed different subcellular localizations (Supplemental Fig. 9). For instance, the full length SIP1::GFP fusion protein and SIP1ΔN::GFP were observed throughout the entire neuron, with preferential localization of the full length SIP1::GFP at the plasma membrane and in the cytosol (Supplemental Fig. 9a-f). In contrast, the SIP1ΔC::GFP fusion proteins were mostly found in the nucleus (Supplemental Fig. 9g–i). We then asked, do any of these three SIP1::GFP fusion proteins retain the function of SIP1 in regulating neurogenesis? As expected, overexpression of the full-length SIP1::GFP fusion protein could rescue the *Nuwa*-associated neurogenesis defect (Fig. 6h). However, neither SIP1ΔN::GFP nor SIP1ΔC::GFP was capable of rescuing the *Nuwa*-associated neurogenesis phenotype (Fig. 6i,j). Since SIP1 was originally identified as a binding protein of Peanut (Pnut, a *Drosophila* Septin essential for cytokinesis)^21,22^ and only the preferentially plasma membrane/cytosol-localized full-length SIP1::GFP could rescue the *sip1*-deficient neurogenesis phenotype (Fig. 6h), we further examined the possibility that *sip1* could affect mitosis. We therefore generated MARCM clones at NHL and analyzed them at 30 h ALH. Interestingly, we found that the average cell numbers of neuroblast clones were 7.08 ± 3.66 and 3.31 ± 1.44 in wild-type (n = 13) and *sip1* mutants (n = 16, *P* < 0.01), respectively (Supplemental Fig. 10. Moreover, neuroblasts containing a mitosis marker, phospho-Histone H3 (H3-P) were 69% and 25% in these wild-type and *sip1* MARCM clones, respectively. Taken together, these results suggested that both the N- and C-terminal domains of SIP1 protein are essential for SIP1 function in the regulation of neurogenesis. These domains potentially target SIP1 to different subcellular localizations and are possibly important for mitosis.

## Discussion

Neurogenesis is robustly, tightly and plastically regulated by developmental stage-dependent molecular and genetic programs to produce appropriate neuronal populations in the *Drosophila* central brain^4–11^. In this study, our MARCM-based genetic screen revealed a novel mutation, *Nuwa* (loss-of-function in *Drosophila sip1*), which impaired neurogenesis in all examined neural lineages at the early larval stage but not embryonic or late larval stages (Figs. 1, 2, 3), suggesting a developmental stage-dependent effect of *sip1* in *Drosophila* central brain neurogenesis. Intriguingly, when the homozygous *Nuwa* mutation was induced at the embryonic stage, embryonic-born adPNs were produced as normal, but larval-born adPNs derived from the same adPN neuroblast were completely absent (Fig. 3 and Supplemental Fig. 4). Together with the results of our SIP1 expression and RNAi knockdown experiments (Fig. 5), this result implies that the function of *sip1* might somehow be linked to the neurogenesis process just after quiescent adPN neuroblast reactivation. However, it remains unclear whether *sip1* is broadly required in various neural lineages (other than adPNs) at this time-point. It is also unclear how *sip1* affects neurogenesis in the early larval stage, especially during the critical period after quiescent neuroblast reactivation. Future studies involving induction of the *Nuwa* mutation in other neuroblasts at the embryonic stage and identifying the interacting partners of SIP1 might address this issue.

Previously, SIP1 was identified as a binding partner for a *Drosophila* Septin, Pnut, through a yeast-two hybrid screen^21^. Interestingly, like other Septin proteins, Pnut was shown to be localized to the cleavage furrow of dividing cells during cytokinesis, and *pnut* loss-of-function mutations resulted in clusters of large, multi-nucleated cells, possibly due to a failure of cytokinesis^22^. It is well established that Septin filamentous structures formed by a network of multiple Septin proteins (Pnut included) are essential for cytokinesis in dividing cells^22–24^. Although the physical and physiological interactions between SIP1 and Pnut have not yet been demonstrated (other than the yeast two-hybrid result), a hint that SIP1 is potentially linked to cytokinesis was observed in 30 h ALH *sip1* mutant clones. Despite that we cannot rule out the possible role of *sip1* in apoptosis, these *sip1* mutant clones had lower cell numbers and mitosis marker H3-P (Supplemental Fig. 10), which could help to explain the neurogenesis defect observed in adult brains with *sip1* mutant neuroblast clones. However, further investigations will be needed to clarify whether SIP1 participates in cytokinesis during neuroblast proliferation or in apoptosis of neural cells and how such participation might affect neurogenesis.

In contrast to *Drosophila* SIP1, the known characteristics of the mouse homologue of SIP1, Tuftelin-interacting protein (TFIP11), would imply that SIP1 might have a different and perplexing function. In another yeast two-hybrid screen, TFIP11 was identified as a binding partner for Tuftelin, one of the major proteins in enamel biomineralisation and possibly an RNA splicing factor^19^. Since there is no evidence that TFIP11 regulates neurogenesis-related processes to our knowledge, we cannot easily reconcile the functions of SIP1 and TFIP11 in the two systems. Interestingly, functional and developmental studies on the worm homologue of *Drosophila* SIP1, Septin and Tuftelin interacting protein 1 (STIP-1), may provide a solution for this conundrum. First, it is known that SIP1, TFIP11 and STIP-1 are functionally conserved proteins in worms, insects and mammals since embryonic lethality in *C. elegans* lacking *stip1* can be rescued by overexpression of either *Drosophila sip1* or human *tfip11*^13^. Second, STIP-1 is crucial for the early embryonic development and may be linked to cell division since worms with *stip-1* knockdown exhibited arrested development and morphological abnormalities around the 16-cell stage^13^. Besides the STIP-1 studies in *C. elegans*, TFIP11 has also been linked to proliferation of cancer cells, as it was upregulated in non-small cell lung cancer (NSCLC); furthermore, knockdown of TFIP11 expression inhibited NSCLC cell proliferation, possibly due to cell cycle arrest and induction of apoptosis by key cell cycle- and apoptosis-related proteins^25^. Studies on TFIP11 in NSCLC together with the studies of STIP-1 in *C. elegans* may provide clues for deciphering the potential role of SIP1 in *Drosophila* central brain neurogenesis. Intriguingly, by studying RNA splicing co-factors in cell cycle and lineage progression in neuroblasts, *sip1* RNAi knockdown caused underproliferation phenotype (*sip1* was considered as a RNA splicing-related factor in Supplemental Fig. 11 of the Abramczuk study^26^), which is similar to our MARCM experiments on *sip1* mutants at the early larval stage (Supplemental Fig. 10). However, the subcellular distributions of TFIP11 and STIP-1 (in the nucleus of cultured cells) reported in previous studies^13,19^ are very different from the localization of SIP1 (in plasma membrane/cytosol of neural cells) we saw in vivo (Fig. 6b-d and Supplemental Fig. 9). Therefore, future investigations into pre-RNA splicing, transcriptional regulation, apoptosis and Septin-directed cytokinesis will be crucial for elucidating the function and functional localization of SIP1 that control neurogenesis in the early developing larval brain.

## Materials and methods

### Generation of transgenic and *sip1* mutant flies

Standard molecular biology techniques were used to generate *UAS-transgene* constructs, including *CG7236*, *CG11030*, *CG11147*, *CG11149*, *DIP-θ*, *sip1*, *sip1::GFP*, *sip1*ΔC*::GFP* and *sip1*ΔN*::GFP*. *UAS-transgene* constructs and two BAC genomic DNA clones (*CH321-13P21* and *CH321-86B19* obtained from Pacman Resources^16^) were used to generate various transgenic flies by integrating DNA fragments into the VK33 docking site; performed by WellGenetics Inc. The *sip1*^*GCFC*^ mutant fly was generated by using the standard CRISPR-Ca9 method^17,18^ to replace the coding sequence between 1240 and 1293 bp from the ATG site with a DNA fragment carrying the RFP-stop cascade, which should abolish the production of the GCFC domain; performed by WellGenetics Inc.

### Fly strains used in this study

The fly strains used in this study were: (1) *hs-FLP[122]*^27^; (2) *tubP-GAL80,FRT*^*40*^^*A*^ (BDSC5192); (3) *FRT*^*40A*^ (BDSC8212); (4) *GAL4-MZ699*^28^; (5) *UAS-mCD8::GFP*^12^; (6) *P*^*111477*^*,FRT*^*40A*^ (DGRC111477); (7) *P*^*111197*^*,FRT*^*40A*^ (DGRC111197); (8) *P*^*102523*^ (DGRC102523); (9) *P*^*102523*^*,FRT*^*40A*^ (this study); (10) *GAL4-OK107* (BDSC854)*;* (11) *acj6-GAL4*^29^; (12) *GAL4-GH146*^30^; (13) *UAS-rCD2::RFP,UAS-GFPRNAi,FRT*^*40A*^ ^31^; (14) *UAS-mCD8::GFP,UAS-rCD2RNAi,FRT*^*40A*^ ^31^; (15) *Act-FRT* < *stop* < *FRT-GAL4*^3^; (16) *UAS-CG11030[VK33]* (this study); (17) *UAS-DIP-θ[VK33]* (this study); (18) *Df(2L)Exel8016* (BDSC7789); (19) *Df(2L)ED270* (BDSC8039); (20) *CH321-13P21[VK33]* (this study); (21) *CH321-86B19[VK33]* (this study); (22) *UAS-CG7236[VK33]* (this study); (23) *UAS-11147[VK33]* (this study); (24) *UAS-CG11149 [VK33]* (this study); (25) *UAS-sip1[VK33]* (this study); (26) *sip1*^*SY*^ (this study); (27) *sip1::sfGFP* (Vienna Drosophila Resource Center, VDRC318488); (28) *worniu-GAL4* (BDSC56553); (29) *UAS-sip1RNAi[attP40]* (BDSC56933); (30) *synaptobrevin-GAL4* (also called R57C10-GAL4, BDSC39171); (31) *UAS-sip1::GFP[VK33]* (this study); (32) *UAS-sip1*ΔC*::GFP[VK33]* (this study); (33) *UAS-sip1*ΔN*::GFP[VK33]* (this study) and (34) *Ase-GAL4*^32^.

### Clonal analysis with MARCM and twin-spot MARCM

The generation, dissection, immunostaining and mounting of mosaic clones in adult brains have been described^12,33^. For MARCM experiments, mosaic clones were induced from NHL to 96 h ALH by heat-shock for 15–40 min. For twin-spot MARCM experiments, mosaic clones of embryonic-born adPNs were generated by heat-shock for 12 min. Primary antibodies used in this study included rat monoclonal antibody to mCD8 (1:100, Invitrogen), chicken antibody to GFP (1:800, Invitrogen), rabbit antibody to GFP (1:800, Invitrogen), rabbit antibody to RFP (1:800, Clontech), rabbit antibody to γ-aminobutyric acid (GABA; 1:100, Sigma), rabbit antibody to phospho-Histone H3 (H3-P; 1:200, Millipore), nc82 (1:100, Developmental studies hybridoma bank/DSHB) and mouse antibody to choline acetyltransferase (Chat; 1:100, DSHB). Secondary antibodies with different fluorophores, including Alexa 488, 546 and 647 (Invitrogen), were used at 1:800 dilutions in this study. Immunofluoroscence images were collected by Zeiss LSM 700 or 780 confocal microscopy and further processed using Adobe Photoshop. The plugin “Cell Counter” and the analyzing tool “ROI Manager” from Fiji ImageJ were used to count neuronal number and estimate the SIP1::sfGFP expression, respectively. One-way ANOVA with post-hoc Tukey test was used for statistical analysis in this study.

## Supplementary Information

Supplementary Information.
