# Supplementary material for: Drosophila septin interacting protein 1 regulates neurogenesis in the early developing larval brain
Source: Sci Rep. 2022 Jan 7;12:292. doi: 10.1038/s41598-021-04474-3 (PMC8742078; doi:10.1038/s41598-021-04474-3)
Supplement: Supplementary file 1 — Supplementary Information. [file 41598_2021_4474_MOESM1_ESM.pdf]

Drosophila septin interacting protein 1 Regulates Neurogenesis in the Early Developing Larval Brain

Jia-Yi Wei, Sao-Yu Chu, Yu-Chien Huang, Pei-Chi Chung and Hung–Hsiang Yu*

Institute of Cellular and Organismic Biology, Academia Sinica, Taipei, Taiwan.

*Corresponding author

E-mail: samhhyu@gate.sinica.edu.tw

SUPPLEMENTARY INFORMATION

Cell number of vPNs in MARCM clones of Figure 1

wt, NHL-24hALH (A)	*111477*, NHL-24hALH (B)	*wt*, 48hALH (C)	*111477*, 48hALH (D)	*wt*, 72hALH (E)	*111477*, 72hALH (F)	*wt*, 96hALH (G)	*111477*, 96hALH (H)
60	5	52	24	32	32	26	24
66	5	54	22	29	27	18	20
68	3	59	16	31	29	19	17
58	5	55	23	28	26	16	18
72	5	51	22	29	17	28	17
	3	49	19	32	29		
	2	48					
	4	46					
	4	55					
	3						
	5						
	4						
	4						
	7						

One-way ANOVA with post-hoc Tukey test

treatments pair	Tukey HSD Q statistic	Tukey HSD p-value	Tukey HSD inferfence
(A) vs (B)	45.6049	0.0010053	** p < 0.01
(C) vs (D)	23.1492	0.0010053	** p < 0.01
(E) vs (F)	2.3774	0.6755792	insignificant
(G) vs (H)	1.3641	0.8999947	insignificant
(A) vs (C)	8.9214	0.0010053	** p < 0.01
(C) vs (E)	16.3285	0.0010053	** p < 0.01
(E) vs (G)	5.6776	0.0047604	* p < 0.01
(B) vs (D)	13.4907	0.0010053	** p < 0.01
(D) vs (F)	3.8491	0.1407480	insignificant
(F) vs (H)	4.8357	0.0260580	* p < 0.05

Supplemental figure 1. Statistical analysis of vPN neurogenesis in wild-type and P^{111477} mutant samples.

Cell numbers of vPNs of wild-type and P^{111477} mutant samples in MARCM clones of Figure 1 are shown in the left panel. The results of one-way ANOVA with post-hoc Tukey's test between samples are shown in the right panel.

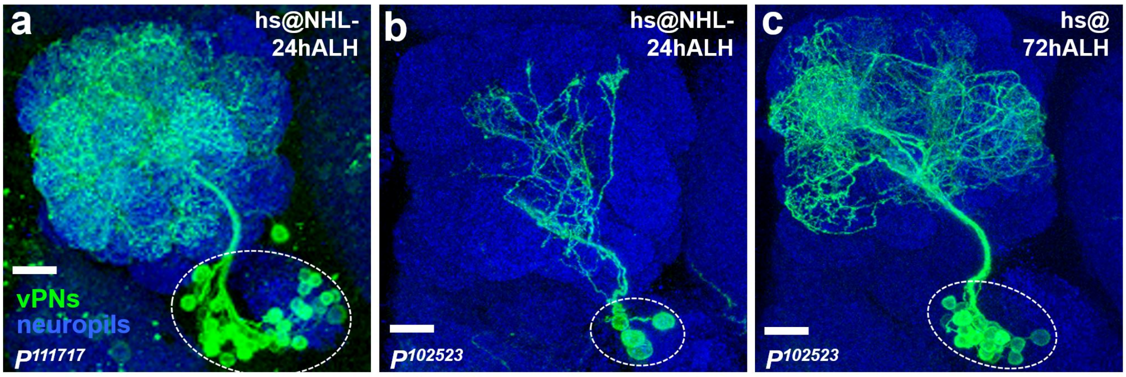

d

Cell number of vPNs in MARCM clones

wt, NHL-24hALH (A)	*102523*, NHL-24hALH (B)	*wt*, 72hALH (C)	*102523*, 72hALH (D)
60	5	32	26
66	4	29	27
68	4	31	19
58	2	28	
72	4	29	
	3	32	

One-way ANOVA with post-hoc Tukey test

treatments pair	Tukey HSD Q statistic	Tukey HSD p-value	Tukey HSD inferfence
(A) vs (B)	38.2815	0.0010053	** p < 0.01
(C) vs (D)	3.4614	0.1104083	insignificant
(B) vs (D)	10.9785	0.0010053	** p < 0.01

Supplemental figure 2. P^{102523} but not P^{111717} mutants exhibited the vPN neurogenesis defect.

(a) The P^{111717} insertion line (*Fas3* mutation) did not have a neurogenesis defect in vPNs when MARCM clones were induced at NHL-24h ALH. (b,c) The P^{102523} insertion line recapitulated the vPN neurogenesis defects observed in the P^{111477} insertion line when MARCM clones were induced at NHL-24h ALH and 72h ALH. Neuropils were revealed by Brp staining (blue). Scale bar: 10 μm. (d) Cell numbers of vPNs in wild-type and P^{102523} mutant samples in MARCM clones are shown in the left panel. The results of one-way ANOVA with post-hoc Tukey's test between samples are shown in the right panel.

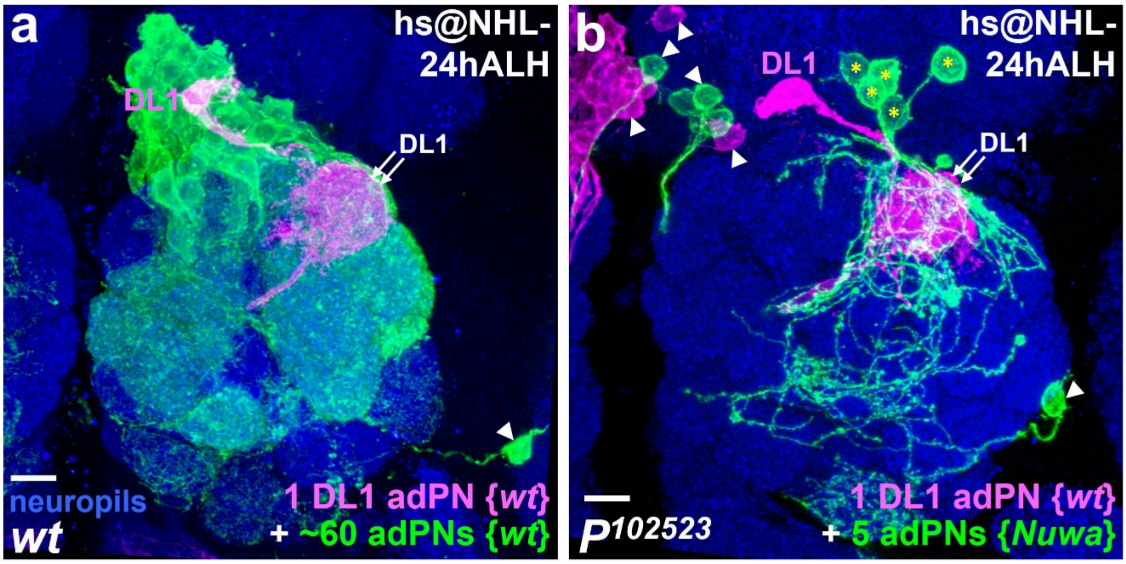

Supplemental figure 3. Neurogenesis of adPNs was significantly impaired when the *Nuwa* mutation was induced at the early larval stage.

(a,b) adPNs displayed severe neurogenesis defects in the *Nuwa* mutants compared to wild types when twin-spot MARCM clones were induced at NHL-24h ALH using *acj6-GAL4*. In the wild-type sample (panel a), a DL1 adPN (the first larval-born adPN type, shown in magenta) was associated with around 60 adPNs (larval-born adPNs, shown in green). In the P^{102523} mutant (panel b), a DL1 wild-type adPN (magenta) was associated with five *Nuwa* mutant adPNs (green, marked by asterisks). Neuropils were revealed by Brp staining (blue), and background neurons are indicated by arrowheads. Scale bar: 10 µm.

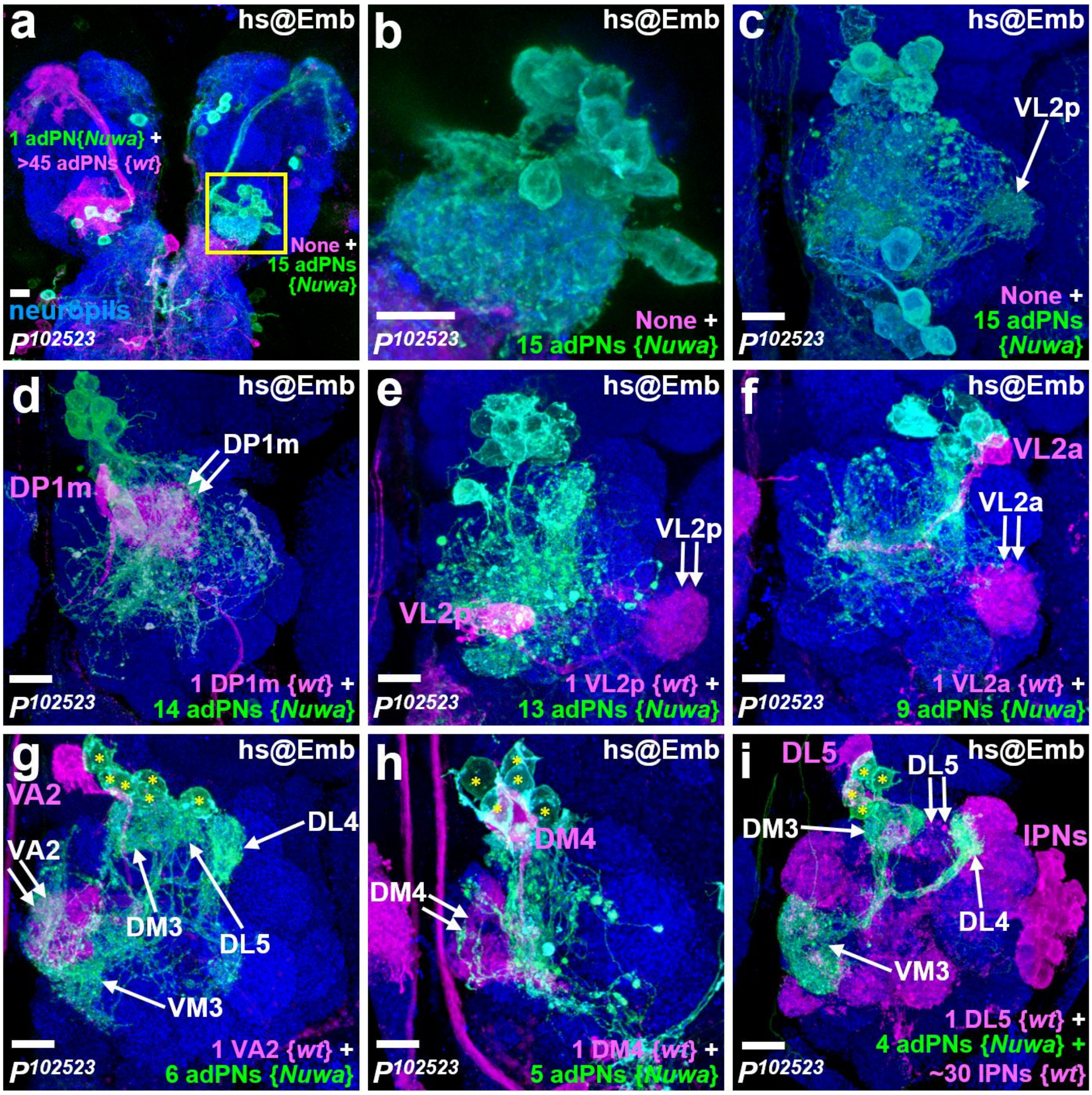

Supplemental figure 4. Twin-spot MARCM analyses of the *Nuwa* mutation on the adPN neurogenesis when the mosaic clones were induced at the embryonic stage.

(a-i) All other examples of *Nuwa* mutant twin-spot MARCM clones generated in this study are shown, including those examined at the wandering larval stage (a) and adulthood (b-i), when the mosaic clones with P^{102523} mutation were induced at the embryonic stage. Fifteen *Nuwa* mutant adPNs are labeled in green (panels a-c). An embryonic-born wild-type adPN (DP1m, VL2p, VL2a, VA2, DM4 and DL5, labeled in magenta) was associated with

various numbers of *Nuwa* mutant adPNs (14, 13, 9, 6, 5 and 4, respectively, labeled in green in panels b-i). Cell bodies of *Nuwa* mutant adPNs are marked by asterisks in panels g-i. Glomeruli innervated by dendrites of *Nuwa* mutant adPNs are indicated in panels g,i. An extra lPN neuroblast clone was observed in panel i. Neuropils were revealed by Brp staining (blue). Scale bar: 10 μm.

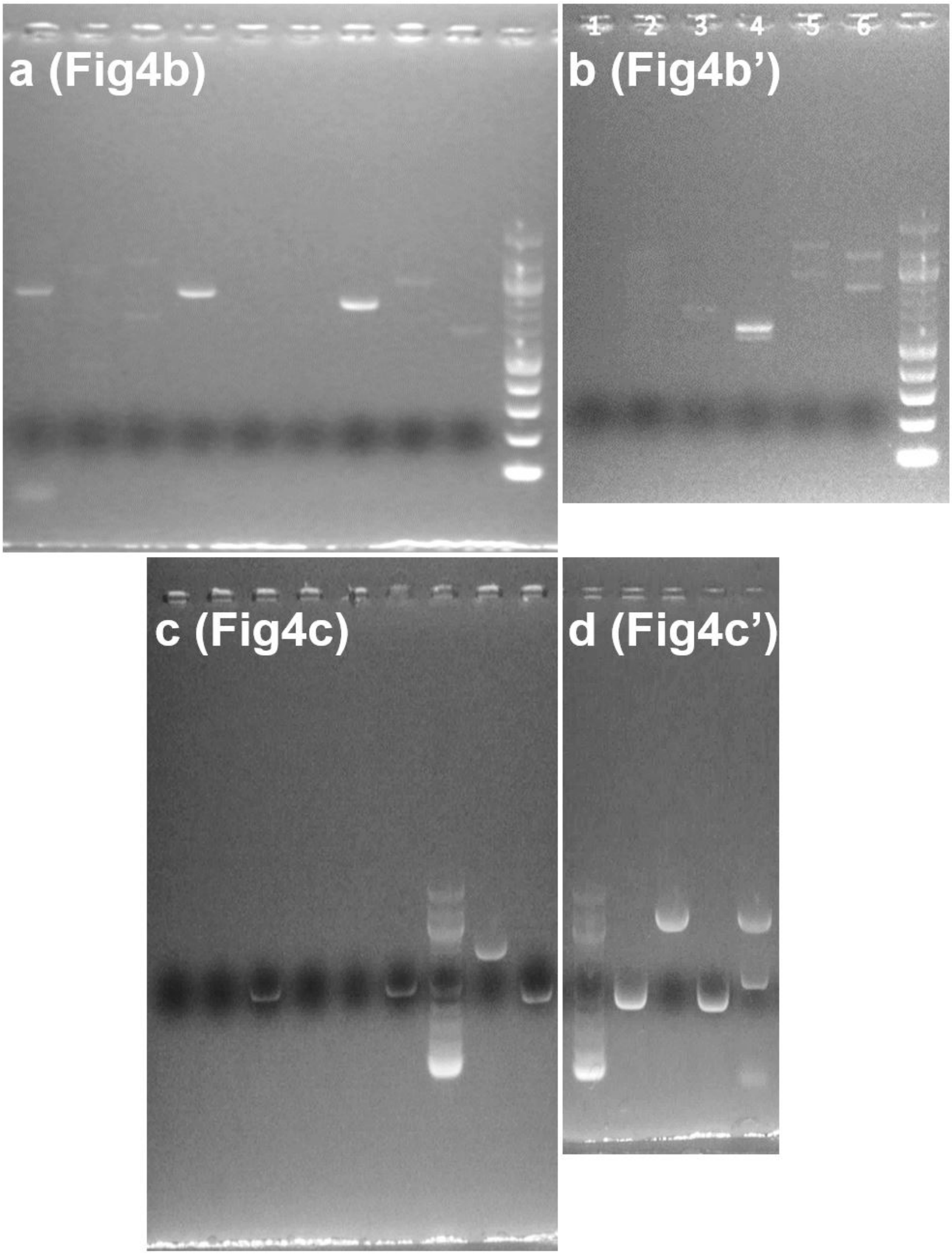

Supplemental figure 5. Original gel images used in Figure 4.

(a-d) Original gel images of Figure 4b, 4b', 4c and 4c' are shown in panels a-d.

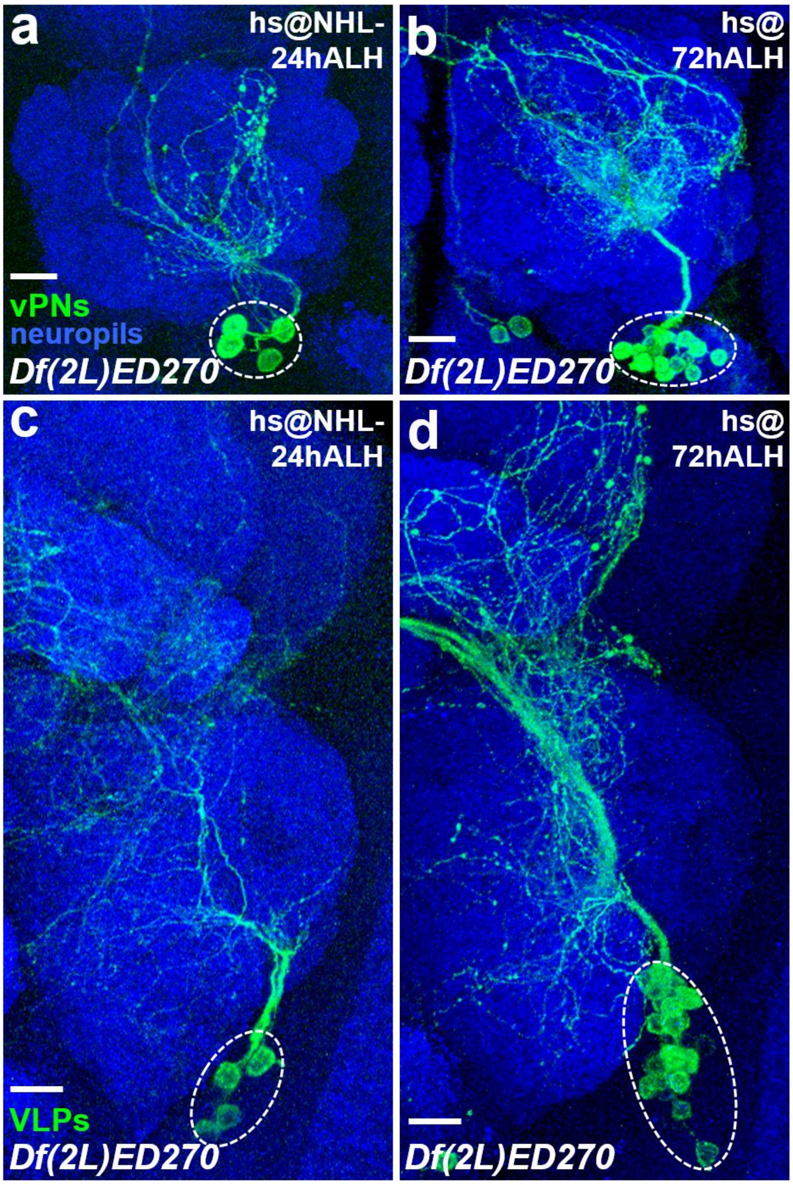

Supplemental figure 6. *Df(2L)ED270* displayed neurogenesis defects similar to the *Nuwa* mutation.

(a-d) The homozygous mutation of *Df(2L)ED270* displayed neurogenesis defects similar to the *Nuwa* mutation in vPNs (panels a,b) and VLPs (panels c,d) when MARCM clones were induced at NHL-24h ALH and 72h ALH. Neuropils were revealed by Brp staining (blue). Scale bar: 10 μm.

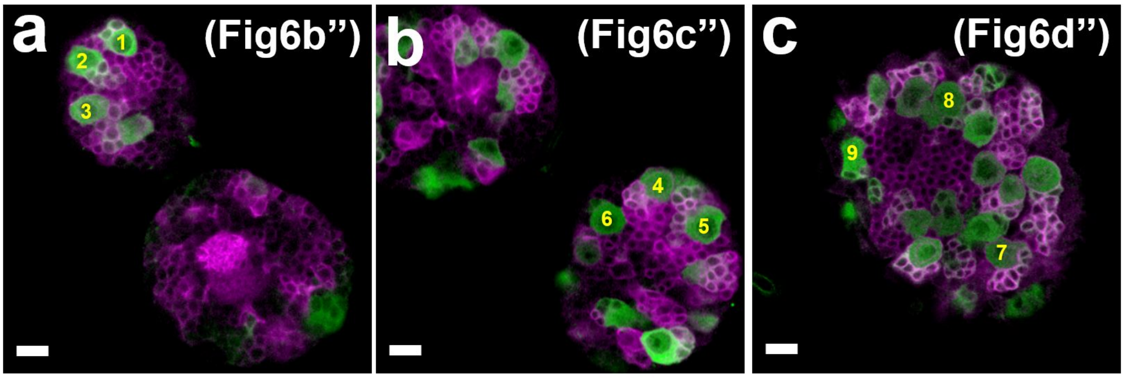

	GFP intersity/area															
	section 1	section 2	section 3	section 4	section 5	section 6	section 7	section 8	section 9	section 10	section 11	section 12	section 13	section 14	section 15	total	ratio
NB1			39/25.8	78.6/27.9	109.6/38.1	131/50	130.8/73.5	137.9/73	141.2/71.7	141.6/78.9	135.3/84.1	110.7/81.8	84.3/75	56.7/63.4	38.7/35.1	1335/778.4	1.72
Ns-1	29/36.1	38.9/59.3	43.1/98.4	54.2/95.4	61.1/96.7	85.4/68.5	82.1/40.1	103/45.4	109.3/39.7	94.8/34.3	44.5/49.1	33.9/16.9				779.2/679.8	1.15
NB2	100/49	108.4/88.6	124.2/96.3	132.9/96	135.3/100.1	137.8/89.2	123.9/61.8	104.1/50.9	78.2/34.9	48.3/25.3	29.3/19.5					914/574	1.59
Ns-2	19.8/50	23/46.7	38.9/44.1	54.6/47.5	53.8/80.4	74.8/100	91.1/103.1	91.1/95.6	75.1/88.3	59.2/86.5	42.8/64.4	26.4/58.8				650.7/865.3	0.75
NB3	32.1/40.3	55.1/53.7	72.6/73	83.7/86.9	90.7/99.6	102.2/104.8	110.2/101.8	108.9/77.4	91.9/47.1	74/40.1	56.8/24.9	36.4/17.3				914.7/766.8	1.19
Ns-3				22.2/59.6	26.2/66.6	36/70.7	41.8/80.9	60.9/83.1	75.5/101.7	78.3/92.9	59.5/109	48.4/100.6	36.5/85.6			485.3/850.9	0.57

	GFP intersity/area															
	section 1	section 2	section 3	section 4	section 5	section 6	section 7	section 8	section 9	section 10	section 11	section 12	section 13	section 14	section 15	total	ratio
NB4	30.9/44.8	49.1/68.5	70.4/93.5	80.2/118.2	89.3/121.9	92/121.2	92.6/109.5	91/97.2	85.1/89.1	88.7/60.3	74.4/38.6	46.7/35.8	30.1/29.6			920.5/1028.2	0.9
Ns-4				46.6/34.7	42.4/68.3	50.1/74	57.1/82.9	60.7/84.5	55/101.3	53/117.3	53.7/111	46/111.9	34.7/95.4	20.9/77.9		520.2/959	0.54
NB5				47.4/27.5	59.1/44.7	69.1/51.7	73.3/70.2	76.1/83.2	78.3/93.7	81.3/100.2	84.8/104.4	85.1/97.6	75.9/92.8	56.6/86.1	28.6/86.1	815.6/937.9	0.87
Ns-5	14.7/67.5	22.1/63.8	23.3/104.8	24.8/112.2	31.6/109.9	38.3/77.3	48.1/83.2	51.4/85.8	50.5/53.3	44.7/35	27.8/16.5					377.3/809.2	0.47
NB6	32.7/60.6	53.9/59.6	65.9/61.6	73.2/87.9	73.4/88.2	78.9/90.7	81.8/99.3	80.7/101.1	80.7/80.7	71.6/68.2	31.5/37					724.5/834.9	0.87
Ns-6	32/37.5	34.3/63.2	45.4/68.9	51.4/71.8	55/77.6	50.6/73.5	46.4/84.4	48.6/77.7	43/78.2	35.8/88.1	32.8/80	27.4/66				502.9/866.9	0.58

	GFP intersity/area															
	section 1	section 2	section 3	section 4	section 5	section 6	section 7	section 8	section 9	section 10	section 11	section 12	section 13	section 14	section 15	total	ratio
NB7				27.3/37	38.7/43.5	43.6/58.6	47.5/79.3	49.9/88.5	53.4/88.1	55.1/94.9	59/87.3	62.5/79.2	65.3/69.1	55.9/61	28.4/46.5	586.6/833.1	0.7
Ns-7	16.9/81.2	17.9/77.6	21.4/89.7	23.6/91.5	25.5/104.1	26.4/169.6	33.3/166.2	35.9/134	41.7/105.5	47.7/101.3	46.9/94.8	43.9/92.8	42.8/78.7	33.4/41.4		457.2/1428.3	0.32
NB8	24.8/34.6	42.2/46.4	50.2/65.2	54.2/112.8	57.2/116.7	63/116.5	67/108.7	64.6/98.3	67.7/76.4	63.5/62.1	49.7/41.9	47/15.3				651.1/894.8	0.73
Ns-8	26/28.4	34.7/25.8	35.7/23.5	40.6/26.6	38.6/25.6	26.7/104.8	30.3/105.6	31/132	33.7/132.4	31.8/152.1	31.3/136.1	31.8/107.2	20.6/73.5			412.8/1073.6	0.38
NB9				40.8/16.2	73.7/40.1	94.6/58.7	99/74.6	101.4/82.1	107.7/98.3	110.4/95.9	112/88.2	114.1/75.5	101.8/51.7	68.6/30.7	45/4.9	1069/716.8	1.49
Ns-9	27.9/75.1	27.4/89.2	33.2/79.7	39/90.5	45.7/91.2	50.7/80.4	54/94	62.2/83.4	76.3/60	81.3/53.1	87/47.3	89.6/33.9	89.5/34.9	89.9/19.1	64.4/21.9	918.1/953.6	0.96

Supplemental figure 7. Estimation of the relative SIP1::sfGFP expression level in neuroblasts and neurons.

(a-c) Neuroblasts (NBs) and surrounding neurons (Ns) for measuring the SIP1::sfGFP-expression are indicated in images of Figure 6b’’ (panel a), 6c’’ (panel b) and 6d’’ (panel c). GFP intensity and area for neuroblasts and neurons were quantified in a series of confocal sections. The SIP1::sfGFP-expression was generally higher in neuroblasts than neurons. Scale bar: 10 μm.

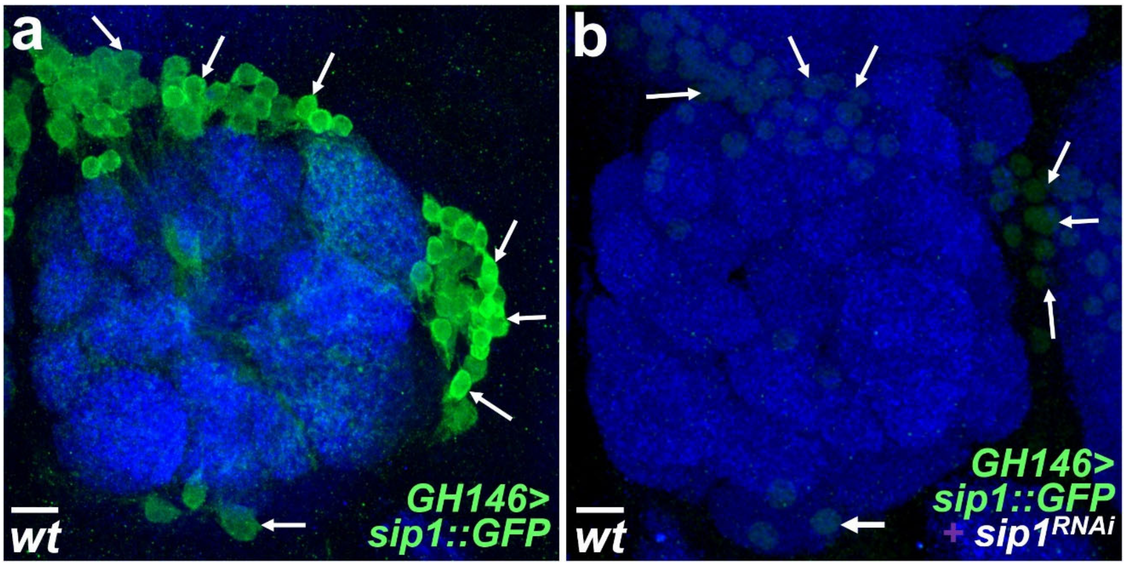

Supplemental figure 8. ***sip1*** **RNAi efficiently blocked SIP1::GFP expression.**

(a,b) SIP1::GFP expression (green in panel a) driven by *GAL4-GH146* was efficiently blocked by overexpression of *sip1* RNAi (arrows in panel b). Neuropils were revealed by Brp staining (blue). Scale bar: 10 μm.

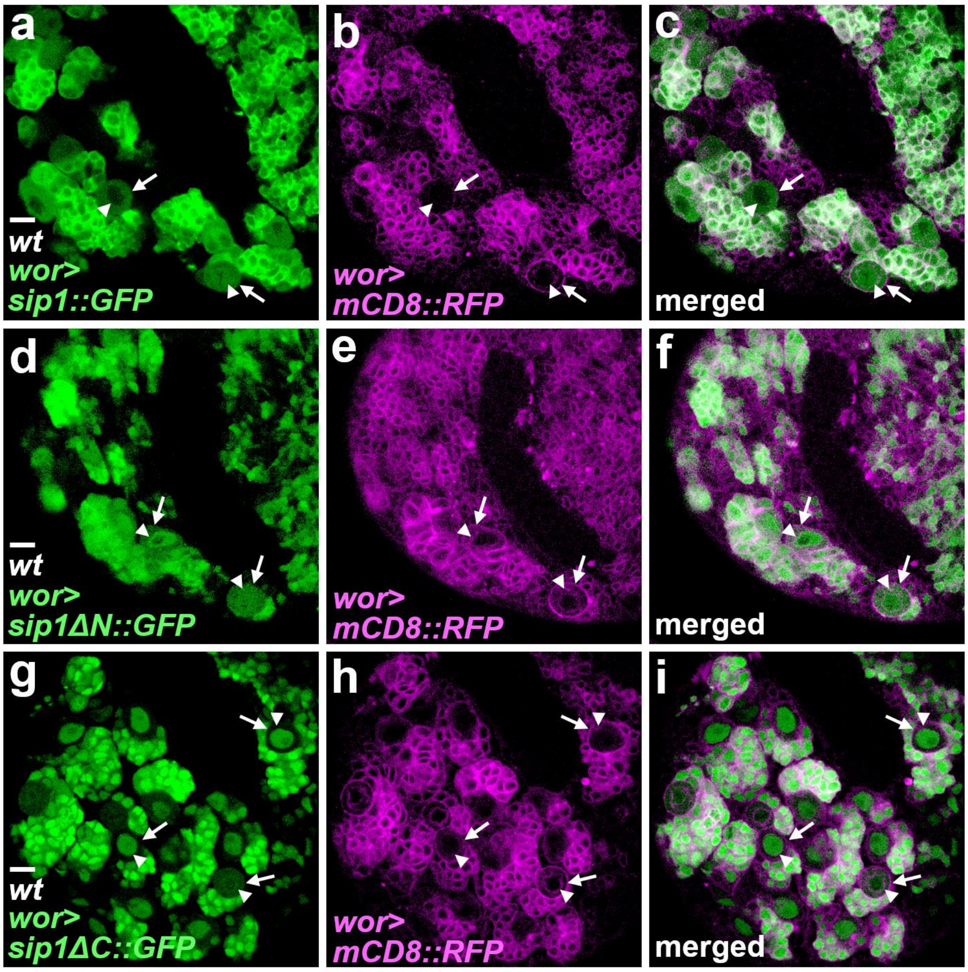

Supplemental figure 9. Full-length and truncated SIP1 proteins displayed different subcellular localizations.

(a-i) The expression of SIP1::GFP (panels a-c), SIP1ΔN::GFP (panels d-f) and SIP1ΔC::GFP (panels g-i) driven by *worniu-GAL4* (*wor*) was examined in developing brains at the mid-late larval stage. The plasma membrane (outer) and the nuclear membrane (inner) of neuroblasts are indicated by arrows and arrowheads, respectively (labeled by mCD8::RFP in magenta). SIP1ΔN::GFP was preferentially expressed at the plasma membrane and in the cytosol (panels a-c). In contrast, SIP1ΔN::GFP was expressed in the whole neuron (panels d-f), whereas SIP1ΔC::GFP was enriched in the nucleus (panels g-i). Scale bar: 10 μm.

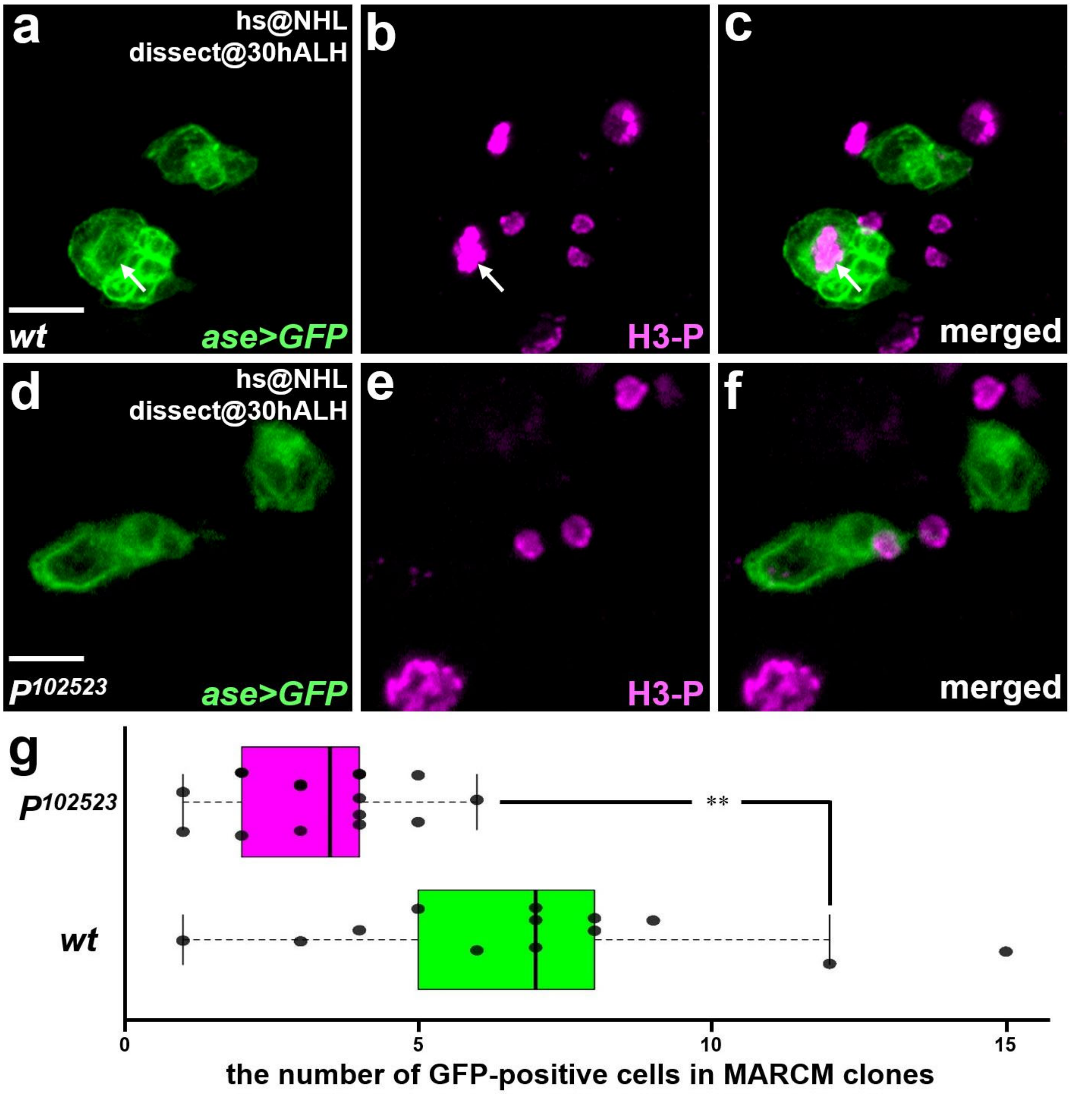

Supplemental figure 10. Cell numbers of wild-type and *sip1* mutant MARCM clones at the early larval stage.

(a-f) MARCM clones in wild-type (panels a-c) and *sip1* (P^{102523}) mutant (panels d-f) samples were generated at NHL, and cell numbers (green) were analyzed at 30h ALH. A mitosis marker phospho-Histone H3 (H3-P; magenta) was also examined for its appearance in neuroblasts of wild-type (69%; arrow) and *sip1* mutant (25%) MARCM clones. Scale bar: 10 µm. (g) Cell numbers of MARCM clones were shown for wild-type (a-c) and *sip1* mutants (d-f).

Supplemental table 1. Genotypes of flies shown in each figure panel

Figure	Genotype
1a, 1d-1g, 2b-2c, 2j-2k	*hs-FLP^{122}/+;FRT^{40A}/tubP-GAL80,FRT^{40A};UAS-mCD8::GFP,GAL4-MZ699/+;+*
1h-1k	*hs-FLP^{122}/+;P^{111477},FRT^{40A}/tubP-GAL80,FRT^{40A};UAS-mCD8::GFP,GAL4-MZ699/+;+*
2d-2e, 2l-2m, S2b-S2c	*hs-FLP^{122}/+;P^{102523},FRT^{40A}/tubP-GAL80,FRT^{40A};UAS-mCD8::GFP,GAL4-MZ699/+;+*
2f-2g	*hs-FLP^{122}/+;FRT^{40A}/tubP-GAL80,FRT^{40A};UAS-mCD8::GFP/+; GAL4-OK107+*
2h-2i	*hs-FLP^{122}/+;P^{102523},FRT^{40A}/tubP-GAL80,FRT^{40A};UAS-mCD8::GFP/+; GAL4-OK107+*
3b	*hs-FLP^{122}/+;UAS-mCD8::GFP,UAS-rCD2RNAi,FRT^{40A}/UAS-rCD2::RFP,UAS-GFPRNAi,FRT^{40A},GAL4-GH146;+;+*
3c, S4a-S4i	*hs-FLP^{122}/+;P^{102523},UAS-mCD8::GFP,UAS-rCD2RNAi,FRT^{40A}/UAS-rCD2::RFP,UAS-GFPRNAi,FRT^{40A},GAL4-GH146;+;+*
5a	*hs-FLP^{122}/+;UAS-mCD8::GFP,UAS-rCD2RNAi,FRT^{40A}/tubP-GAL80,FRT^{40A};Act-FRT<stop<FRT-GAL4/+;+*
5b	*hs-FLP^{122}/+;P^{102523},UAS-mCD8::GFP,UAS-rCD2RNAi,FRT^{40A}/tubP-GAL80, FRT^{40A};Act-FRT<stop<FRT-GAL4/+;+*
5c-5j	*hs-FLP^{122}/+;P^{102523},UAS-mCD8::GFP,UAS-rCD2RNAi,FRT^{40A}/tubP-GAL80, FRT^{40A};Act-FRT<stop<FRT-GAL4/UAS-transgene* or *Pacman BAC;+*
5k-5l	*hs-FLP^{122}/+;sip1^{GCFC},FRT^{40A}/tubP-GAL80,FRT^{40A};UAS-mCD8::GFP,GAL4-MZ699/+;+*
6b-6d, S7a-S7c	*w;wor-GAL4/+;UAS-mCD8::RFP/sip1::sfGFP;+*
6e	*w;+;+;+*
6f	*w;UAS-sip1RNAi/wor-GAL4;+;+*
6g	*w;UAS-sip1RNAi/+;syb-GAL4;+;+*
6h	*hs-FLP^{122}/+;P^{102523},UAS-mCD8::GFP,UAS-rCD2RNAi,FRT^{40A}/tubP-GAL80,FRT^{40A};*

	Act-FRT<stop<FRT-GAL4/ UAS-sip1::GFP;+
6i	hs-FLP^{122}/+;P^{102523},UAS-mCD8::GFP,UAS-rCD2RNAi,FRT^{40A}/tubP-GAL80,FRT^{40A}; Act-FRT<stop<FRT-GAL4/ UAS-sip1ΔN::GFP;+
6j	hs-FLP^{122}/+;P^{102523},UAS-mCD8::GFP,UAS-rCD2RNAi,FRT^{40A}/tubP-GAL80,FRT^{40A}; Act-FRT<stop<FRT-GAL4/ UAS-sip1ΔC::GFP;+
S2a	hs-FLP^{122}/+;P^{111717},FRT^{40A}/tubP-GAL80,FRT^{40A};UAS-mCD8::GFP,GAL4-MZ699/+;+
S3a	hs-FLP^{122}/acj6-GAL4;UAS-mCD8::GFP,UAS-rCD2RNAi,FRT^{40A}/UAS-rCD2::RFP,UAS-GFPRNAi,FRT^{40A};+;+
S3b	hs-FLP^{122}/acj6-GAL4;P^{102523},UAS-mCD8::GFP,UAS-rCD2RNAi,FRT^{40A}/UAS-rCD2::RFP,UAS-GFPRNAi,FRT^{40A};+;+
S6a-S6d	hs-FLP^{122}/+;Df(2L)ED270,FRT^{40A}/tubP-GAL80,FRT^{40A};UAS-mCD8::GFP,GAL4-MZ699/+;+
S8a	w;GAL4-GH146/+;UAS-sip1::GFP/+;+
S8b	w;GAL4-GH146/UAS-sip1RNAi;UAS-sip1::GFP/+;+
S9a-S9c	w;wor-GAL4/+;UAS-sip1::GFP/+;+
S9d-S9f	w;wor-GAL4/+;UAS-sip1ΔN::GFP/+;+
S9g-S9i	w;wor-GAL4/+;UAS-sip1ΔC::GFP/+;+
S10a-S10c	hs-FLP^{122}/+;UAS-mCD8::GFP,UAS-rCD2RNAi,FRT^{40A}/tubP-GAL80,FRT^{40A};Ase-GAL4/+;+
S10d-S10f	hs-FLP^{122}/+;P^{102523},UAS-mCD8::GFP,UAS-rCD2RNAi,FRT^{40A}//tubP-GAL80,FRT^{40A}; Ase-GAL4/+;+

Supplemental table 2. Complementation test for lethality among *Nuwa*-related mutations

Genetic crosses	Curly wing	Straight wing	Complement on lethality
P^{111477},FRT^{40A}/*Cyo* X P^{111477},FRT^{40A}/*Cyo*	124	0	no
P^{102523},FRT^{40A}/*Cyo* X P^{102523},FRT^{40A}/*Cyo*	135	0	no
P^{102523}/*Cyo* X P^{111477},FRT^{40A}/*Cyo*	185	0	no
P^{102523}/*Cyo* X P^{102523},FRT^{40A}/*Cyo*	147	0	no
P^{102523}/*Cyo* X P^{102523}/*Cyo*	119	0	no
Df(2L)Exel8016/Cyo X P^{102523},FRT^{40A}/*Cyo*	123	80	yes
Df(2L)Exel8016/Cyo X P^{102523}/*Cyo*	116	69	yes
Df(2L)ED270/Cyo X P^{102523},FRT^{40A}/*Cyo*	141	0	no
Df(2L)ED270/Cyo X P^{102523}/*Cyo*	103	0	no
$sip1^{GCFC}$/*Cyo* X P^{102523},FRT^{40A}/*Cyo*	224	0	no

Supplemental table 3. Oligos used in Figure 4

Oligo name	Oligo sequence	Note
Plac4	actgtgcgttaggtcctgttcattgtt	Fig2B lanes 1-9
Plac1	cacccaaggctctgctcccacaat	Fig2B lanes 1, 4 and 7
GD-R	cactttactaggtacggcatctg	Fig2B lanes 2, 5 and 8
Sp1	acacaacctttcctctcaacaa	Fig2B lanes 3, 6 and 9;<br>Fig3C lanes 1, 4 and 7
Pry4	caatcatatcgctgtctcactca	Fig2B' lanes 10-15
Pry1	ccttagcatgtccgtggggtttgaat	Fig2B' lanes 10, 12 and 14
Plw3	tgtcggcgtcatcaactcc	Fig2B' lanes 11, 13 and 15
Fas3-5'	ggctttactttcctctgagccgttg	Fig3C lanes 1, 4 and 7
Pry2	cttgccgacgggaccaccttatgttatt	Fig3C lanes 2, 3, 5, 6 and 8;<br>Fig3C' lanes 9-12
Fas3-3'	ttcctagcacccacccaattccctg	Fig3C lanes 2, 5 and 8
DIP-θ	cagccatttaatgagccacgctcgac	Fig3C lanes 3 and 6;<br>Fig3C' lanes 9 and 11
CG11030	ggcagggtggaatatcattgaattgg	Fig3C' lanes 10 and 12
